# Physics‐Based AI‐Augmented Workflows Discovering Candidate Graphene Quantum Dot Near‐Infrared Chromophores

**DOI:** 10.1002/jcc.70522

**Published:** 2026-11-02

**Authors:** Naga Mani Gajula, Muhammad Hammad Ali, Md. Masud Rana, Benjamin G. Janesko

**Affiliations:** ^1^ Department of Computer Science Lamar University Beaumont Texas USA; ^2^ Department of Chemistry & Biochemistry Texas Christian University Fort Worth Texas USA

**Keywords:** fusion‐based hexagonal lattice growth, GFN2‐xTB, graphene quantum dots, machine learning, near‐infrared emission, TD‐DFT

## Abstract

Graphene quantum dots (GQDs) exhibit optical properties that depend strongly on their size, edge structure, and surface functionalization, but identifying the molecular structures responsible for near‐infrared (NIR) emission remains challenging because of their structural complexity and disorder. In this work, we developed a computational framework combining rule‐based molecular generation, semi‐empirical quantum chemistry calculations, machine learning, and time‐dependent density functional theory (TD‐DFT) to predict possible chemically realistic candidate GQDs with NIR‐emitting optical properties. A fusion‐based hexagonal lattice growth approach was used to generate 100,000 candidate graphitic structures. A random subset of 5000 structures was selected for semiempirical calculations. Computed band gaps were used to train and evaluate five machine learning models (Random Forest, XGBoost, LightGBM, CatBoost, and Neural Network). LightGBM predicted bandgaps were used to pre‐screen the remaining 95,000 structures. 11,000 structures with low predicted gaps were selected for semiempirical calculations, and 50 top candidates with low gap and low SA score were selected for TD‐DFT calculations employing LC‐*ω*PBE functional. All 50 structures were predicted to exhibit NIR emission, offering a computed database of candidate NIR‐emitting GQDs for future validation against experiment.

## Graphical abstract

Identifying molecular structures responsible for near‐infrared (NIR) emission in graphene quantum dots (GQDs) remains challenging because of their structural complexity. Here, we develop a computational framework that integrates rule‐based molecular generation, semi‐empirical quantum chemistry calculations, machine learning, and time‐dependent density functional theory (TD‐DFT) to identify chemically realistic candidate GQDs with NIR‐emitting optical properties.

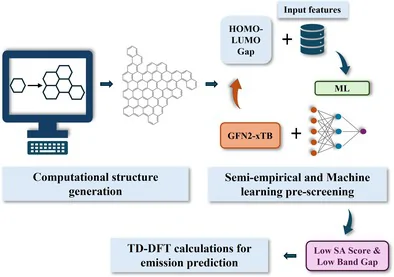

## 1 Introduction

Graphene quantum dots (GQDs) are graphitic nanomaterials possessing sizes around 1–10 nm [1, 2, 3, 4, 5]. Recently synthesized GQDs show promise for bioimaging applications [6]. These GQDs combine high biocompatibility [7], high water solubility [8], substantial photostability [9], and absorption and emission in the near‐infrared (NIR) window [10] desirable for through‐tissue imaging [11]. Both top‐down and bottom‐up syntheses have yielded biocompatible NIR‐emitting GQDs. Photoluminescence properties of GQDs are known to be influenced by quantum confinement effects based on the size and number of graphene layers as well as defect states [12, 13, 14, 15, 16]. While NIR emission is typically assigned to defect sites [4, 12, 17], the chemical structure of GQD NIR chromophores is not known, hampering efforts at further development of GQDs for NIR bioimaging.

We are involved in an ongoing project to identify and characterize GQD NIR chromophores. In the first stage of this project, we assembled a database of candidate chromophores: 16 well‐defined synthetic graphitic nanostructures possessing NIR emission [18]. The structures exhibited extended π‐conjugation, charge‐transfer character, open‐shell diradical character, and/or aggregation‐induced bathochromic shifts. We also calibrated a computational protocol for screening candidate NIR chromophores, combining TD‐DFT simulations of absorption and emission with a synthetic accessibility score [19] (SA score) for initial screening of thermochemical stability.

This manuscript details the next stage of our work. Building on the experimentally determined NIR chromophores and computational workflows collected in ref. [19], we generate an *in silico* database of hypothetical GQD NIR chromophore structures. We present a new computational database of 50 candidate chromophores screened from 100,000 initial candidates, all of which are predicted to combine NIR emission and realistic chemical stability. The screening process combined semiempirical electronic structure calculations with the creation and parameterization of a machine‐learned model predicting graphitic nanostructure band gaps, computed at the semiempirical level. Ultimately, we will use this database to annotate real GQD NIR spectra and develop hypotheses for controlling GQD synthetic conditions to optimize NIR response.

Optical properties of GQDs are influenced by structural features, including size and edge morphology [4, 14]. Defects within the structure and edge imperfections can cause non‐radiative recombination pathways that lead to inefficiencies in the luminescence process [20]. This emphasizes the importance of systematically exploring the molecular structure of GQDs with appropriate electronic properties for NIR emission.

Our work builds on previous efforts in the automated *in silico* generation of chemically meaningful graphitic structures. COMPAS is a database of catacondensed polybenzenoid hydrocarbons with a wide range of possible structural variations [21]; later, the COMPAS‐2 database expanded this database to include heteroatom‐substituted polycyclic aromatic compounds [22]. Stochastic lattice growth techniques have been extensively applied for the simulation of two‐dimensional structure formation. The Eden growth model provided a probabilistic basis for forming compact structures using the sequential attachment of nearby lattice points [23]. Kinetic Monte Carlo models have been developed for the growth of graphene edges, showing that stochastic attachment of carbon atoms can be used to simulate graphene structure formation [24]. Large‐scale kinetic Monte Carlo simulations have been used to further understand the process of graphene growth and etching through hexagonal lattice model representations [25]. Related efforts have involved automated fragmentation of chemical structures. AroBOROS, an aromatic fragmentation method using a ring‐overlap approach, was created to fragment large polycyclic aromatic hydrocarbons (PAHs) into overlapping subsystems of aromatics, allowing for effective computational analysis of their electronic structures [26]. General‐purpose fragmentation software, such as FragIt, has been developed to produce chemical molecular fragments for quantum chemistry calculations [27]. The fusion approach has also been utilized to create large databases of polybenzenoid hydrocarbons (PBHs) [21]. We will use a fusion approach detailed below.

Our work builds on recent literature employing semiempirical electronic structure methods to simulate graphitic nanostructures. Semiempirical methods are attractive for large‐scale simulation as they combine a physically reasonable quantum model with a cost far lower than ab initio or density functional methods [28, 29]. The sTDA‐xTB semiempirical model Hamiltonian provides a reasonable cost:accuracy tradeoff for spectral properties of molecules [29]. The GFN2‐xTB model [28], developed for forces and geometries, has also provided qualitatively reasonable initial guesses for some electronic properties [30]. This work uses GFN2‐xTB HOMO‐LUMO gaps in the initial prescreening.

Our work also builds on an extensive recent literature employing machine learning to predict the results of more expensive quantum chemistry calculations. Machine learning models that are commonly used in such scenarios include Random Forest [31], XGBoost [32], LightGBM [33], and CatBoost [34]. One benchmark study conducted on a scale of 1.4 million chemical compounds revealed that while XGBoost gave the best predictive accuracy in terms of modeling, LightGBM took the shortest time for training [35]. In this study, the SHAP (Shapley Additive Explanation) method has been used to explain the predictions of the models by assessing the impact of each property individually on the predicted values [36]. We also use the RDKit cheminformatics package for structure generation and computation of SA score [19, 37].

In summary, the current structure generation studies have been limited to either catacondensed or heteroatom‐doped topologies that do not involve NIR‐directed screening [21, 22]; while fragmentation procedures have been applied to already known PAHs rather than structure generation [26], and Eden‐growth‐based approaches have been mainly used to model the kinetics of physical growth or etching [23, 24, 25]. Both semiempirical methods and SA scores are useful for large‐scale screening but require validation before they can serve as definitive criteria for predicting optical properties and synthetic viability [19, 28, 37]. To address these research gaps, this study develops an approach that combines Eden‐growth‐based hexagonal lattice generation, GFN2‐xTB screening, band‐gap prediction using machine learning, and validation using TD‐DFT. Using this approach, 100,000 hexagonal GQD structures were generated and screened, and 50 top candidates were selected for NIR emission based on GFN2‐xTB HOMO‐LUMO gaps and SA scores. The overall computational workflow is shown in Figure 1.

**FIGURE 1:**
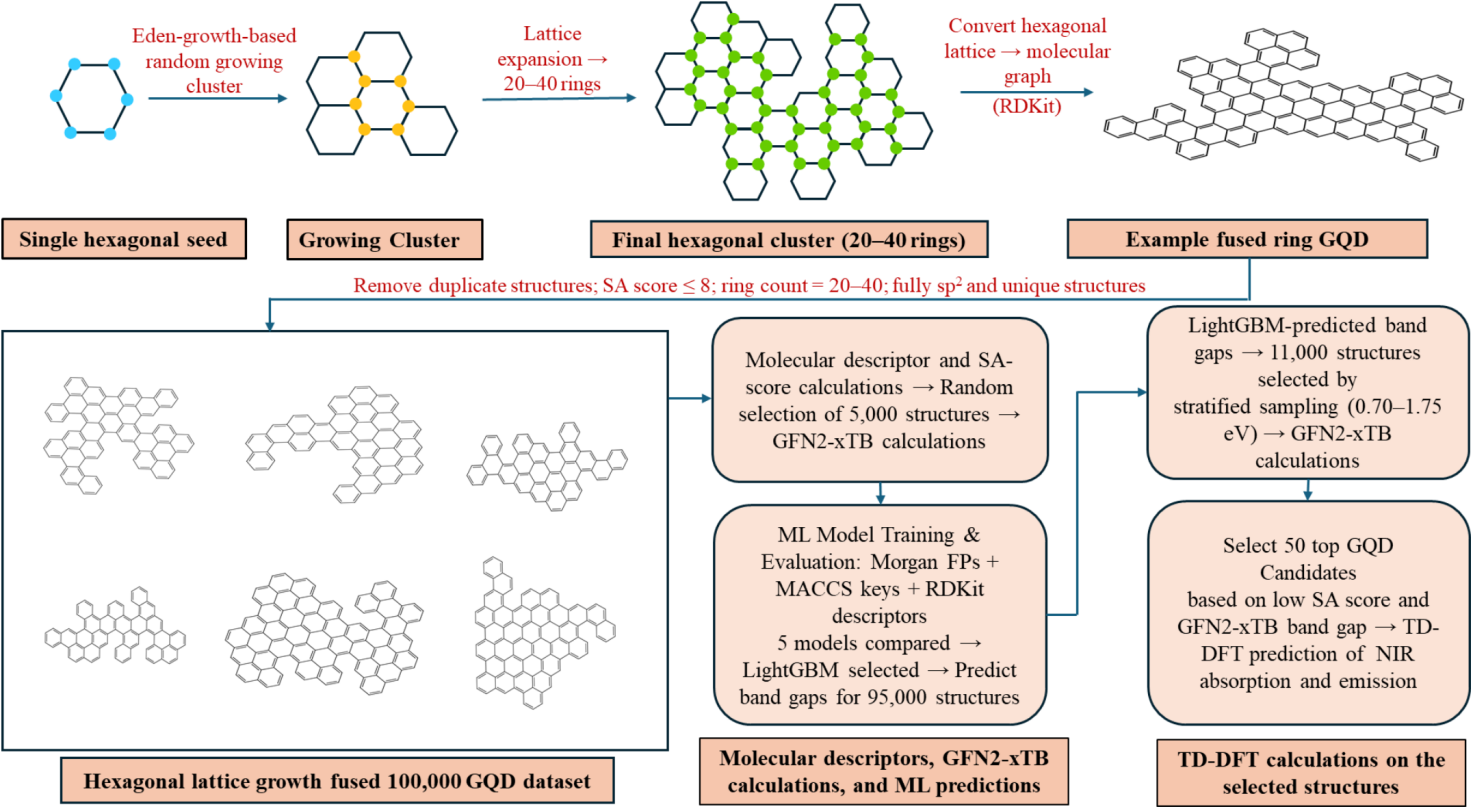
Overall computational workflow for the generation, screening, machine‐learning prediction, and TD‐DFT evaluation of fusion‐based graphene quantum dots (GQDs) with predicted NIR emission. The workflow includes Eden‐growth‐based hexagonal lattice generation, structural filtering, GFN2‐xTB calculations, machine‐learning prediction of HOMO–LUMO gaps, stratified selection of candidates, and TD‐DFT evaluation of the final 50 structures.

## 2 Computational Methods

### 2.1 Hexagonal Lattice‐Based Structure Generation

The structures in this study were generated using a random growth strategy on a hexagonal lattice. This technique allows the development of graphene‐like structures by direct growth from the abstract hexagonal lattice, thereby enabling exploration of wide variety of fused‐ring topologies. The process of growth was carried out on the hexagonal $\left(q,r\right)$ lattice, where each hexagon has six possible sites for growth. A single hexagon was used as the initial seed, and growth proceeded by choosing one site from the growth perimeter until a predefined target size of 20 to 40 fused hexagons was achieved [23]. The Eden growth algorithm grows the structure by randomly selecting a site from the growth perimeter at each step, leading to the formation of a graphene‐like structure.

A molecular graph was generated for each hexagonal cluster by mapping the common carbon atoms to adjacent hexagonal clusters based on their common edges according to the graphene lattice fusion‐ring connection. A standard aromatic C–C bond length of 1.42 Å was used for calculating the two‐dimensional atomic coordinates and defining aromatic bonds of the hexagonal rings. Sanitization of the molecular graph using RDKit ensured proper molecular connectivity, valence, and aromaticity of molecules, and unsanitized molecular graphs were excluded from the dataset. The selected structures were analyzed for other properties as well. The SA score was required to be below 8.0, the percentage of sp^2^ carbon atoms must be greater than or equal to 0.95, and the number of rings had to be between 20 and 40 following the sanitization process. A canonical InChIKey was used to eliminate duplicate structures. The process was repeated until 100,000 compounds met the requirements.

The final dataset required 695,169 growth iterations with a 14.4% acceptance rate. In terms of molecular validation, the majority of the rejected compounds were rejected because RDKit was unable to clean up or kekulize the graph. Only one generated structure was rejected based on the SA score criterion; none failed the sp^2^‐carbon criterion. A random seed 42 was selected for the generation of structures. The final dataset included 100,000 graphene‐like structures, with molecular weights ranging from 716.8 g/mol to 1822.15 g/mol. Molecular weight, LogP, TPSA (topological polar surface area), hydrogen bond donor/acceptor, number of rotatable bonds, QED (quantitative estimate of drug‐likeness), and synthetic accessibility were calculated for each molecule using RDKit. These 100,000 molecules were stored as SMILES strings. SMILES strings were converted to three‐dimensional coordinates using the ETKDG method as implemented in RDKit, and were then preoptimized using the MMFF94 or UFF force fields.

### 2.2 Semiempirical and Machine Learning Predictions of Low Band‐Gap Structures

The next step was to identify promising candidates from the 100,000 generated structures by combining semiempirical quantum chemistry calculations with machine learning. A random subset of 5000 structures was selected for GFN2‐xTB geometry optimizations, and the resulting HOMO‐LUMO gaps were used as the target property for machine‐learning predictions. Five machine learning models (Random Forest, CatBoost, LightGBM, XGBoost, and Neural Network) were trained using molecular features, including Morgan fingerprints, MACCS structural keys (a 167‐bit fingerprint encoding predefined chemical substructures), and RDKit‐derived two‐dimensional molecular descriptors as input features, with the GFN2‐xTB‐calculated HOMO‐LUMO gap as the training label. The LightGBM model was then used to predict the HOMO‐LUMO gaps of the remaining 95,000 generated structures. These band gaps were used to pre‐screen 11,000 structures with low predicted band gaps, which were subsequently evaluated using GFN2‐xTB. Based on the resulting GFN2‐xTB band gaps and SA scores, the top 50 candidates were selected for TD‐DFT calculations.

We calculated Molecular features from the 2D molecular structures using Morgan fingerprints, MACCS structural keys, and RDKit molecular descriptors. Morgan fingerprints were generated at radii of 2, 3, 4, and 5, with 2048 bits for each radius, resulting in a total of 8192 fingerprint features for each structure. We also included 167 MACCS keys and 409 two dimensional molecular descriptors calculated using RDKit, combining these descriptors with the Morgan fingerprints, these features resulted in 8768 molecular features per structure. After cleaning, the molecular library was divided into three groups: 4000 structures were used for model training, 1000 structures were used for model testing, and the remaining structures were used for screening. A random seed of 42 was used for the data split. Before model training, infinite values were treated as missing values and they were replaced with the median of the corresponding feature calculated from the training set. Feature values outside the range of $-10^{6}$ to $10^{6}$ were clipped to this range and then standardized using StandardScaler. The StandardScaler was fitted on the training set and then applied to the test set. We evaluated five regression models: Random Forest, XGBoost, LightGBM, CatBoost, and a multilayer perceptron neural network. The Random Forest model was configured with 200 estimators, a maximum depth of 20, a minimum of 5 samples required for splitting, and a minimum of 2 samples per leaf. XGBoost was configured with 200 estimators, a maximum depth of 7, a learning rate of 0.1, a subsampling ratio of 0.8 for both rows and columns. LightGBM was configured with 200 estimators, a maximum depth of 7, a learning rate of 0.1, 31 leaves, and a subsampling ratio of 0.8 for both rows and columns. CatBoost was configured with 200 iterations, depth of 7, a learning rate of 0.1, and subsample ratio of 0.8. The multilayer perceptron neural network had three hidden layers with 256, 128, and 64 neurons, respectively, using ReLU activation. An initial learning rate of 0.001 was used, with a maximum of 1000 iterations, and early stopping with 10% of the training data used for validation. All models were initialized with a fixed random seed of 42. We assessed model performance using three metrics: the coefficient of determination (R^2^), root mean square error (RMSE), and mean absolute error (MAE). Among the five models, LightGBM achieved the highest test‐set R^2^ (0.6716) and was therefore selected to screen the remaining structures.

### 2.3 TD‐DFT calculations

All density functional theory (DFT) calculations were performed using the Gaussian 16 program package [38]. Ground‐state ($S_{0}$) and first excited‐state ($S_{1}$) geometry optimizations were carried out at the B3LYP/def2‐SVP level of theory. All calculations were performed using spin‐restricted wave functions. Broken‐symmetry calculations were also performed to investigate the possible open‐shell diradical character. Optimized geometries were confirmed as local minima on their respective potential energy surfaces. Vertical excitation ($S_{0}\to S_{1}$) and fluorescence emission ($S_{1}\to S_{0}$) wavelengths were computed within the framework of time‐dependent density functional theory (TD‐DFT) using the linear response formalism with an adiabatic exchange‐correlation (XC) kernel. To evaluate the influence of long‐range exchange on the electronic and photophysical properties of the extended π‐conjugated GQDs, TD‐DFT calculations were performed using both the conventional B3LYP functional and the long‐range corrected LC‐*ω*PBE functional. Unless otherwise specified, all LC‐*ω*PBE calculations employed a fixed range‐separation parameter (ω) of 0.15 Bohr^−1^, which was determined through the Koopmans‐theorem‐based tuning procedure using an experimentally synthesized representative GQD [39].

### 2.4 Optimal Tuning of the Range‐Separation Parameter (ω) of LC‐ω PBE Functional

The electronic and optical properties of extended π‐conjugated systems are highly sensitive to the treatment of long‐range electron–electron interactions. Conventional hybrid functionals often suffer from delocalization and residual self‐interaction errors which can lead to inaccurate excited‐state properties, charge distributions and frontier orbital energies, particularly for highly conjugated systems. To obtain a more accurate description of these systems, the range‐separation parameter (ω) of the LC‐ωPBE functional was optimized using the Koopmans' theorem. The value of ω was varied to minimize the deviation between the HOMO eigenvalue of the neutral system and its ionization potential (IP). The IP was obtained from the energy difference between the neutral ($E_{\mathrm{gs}}\left(\omega ,N\right)$) and corresponding cationic ($E_{\mathrm{gs}}\left(\omega ,N-1\right)$) systems [40]. IP‐optimized ω was obtained by minimizing
(1)

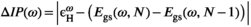




An interpretation of Equation (1) is that, by minimizing $\Delta \mathrm{IP}\left(\omega \right)$, the so‐called many‐electron self‐interaction error (MESIE) in density functionals is minimized which has been identified as one of the principal sources of error in conventional density functional approximations [41]. Consequently, the IP‐optimization of LRC‐hybrids not only improves those properties directly related to the IP, but also solves other issues associated with MESIE [42]. The dependence of ΔIP on ω for the representative GQD employed in the tuning procedure is shown in Figure 2.

**FIGURE 2:**
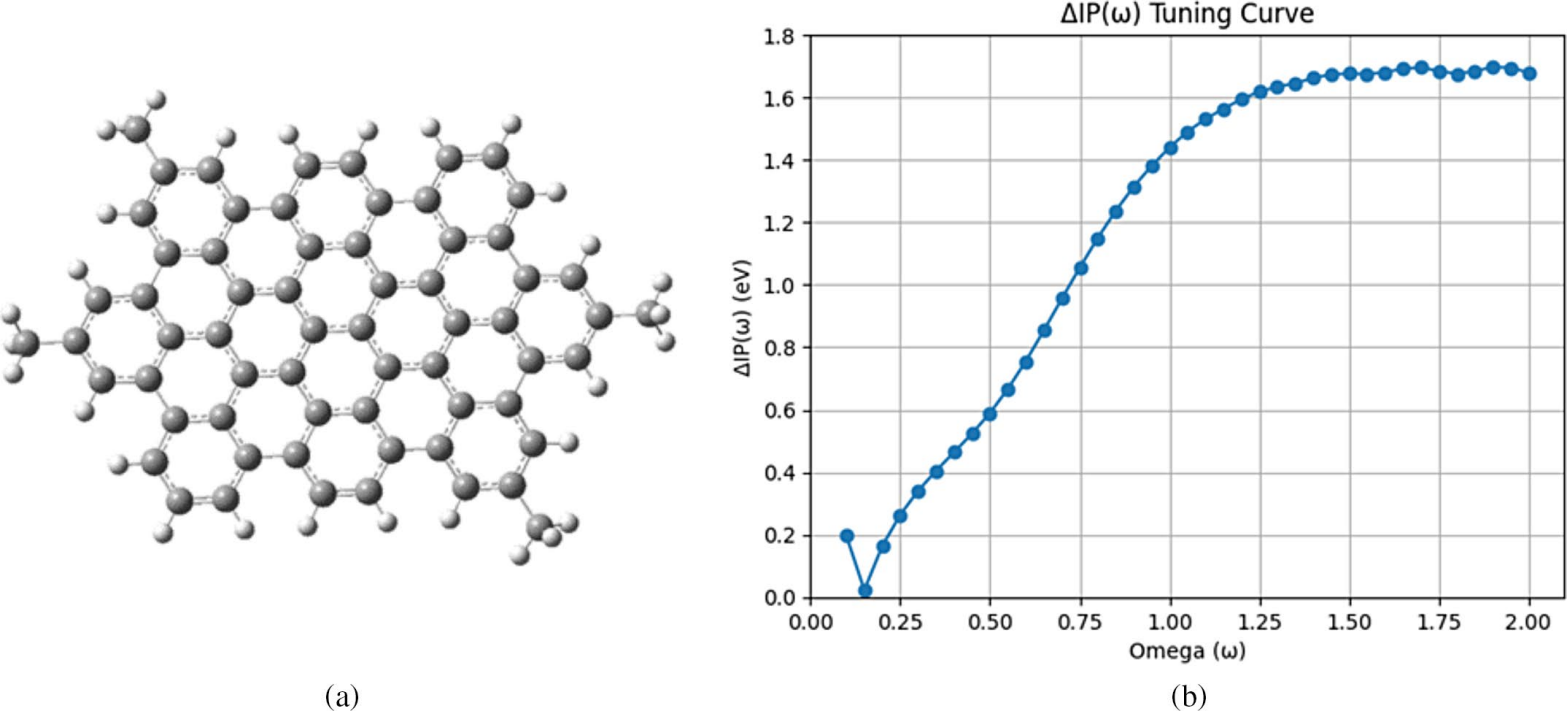
(a) Representative graphene quantum dot (GQD) employed for the optimization of the range‐separation parameter (ω) of the LC‐ωPBE functional. (b) $\Delta \mathrm{IP}\left(\omega \right)$ tuning curve used to determine the optimal ω value according to Koopmans' theorem.

A series of ω values was examined, and the optimal range‐separation parameter (ω) value was identified as 0.15 Bohr^−1^, corresponding to the minimum deviation between HOMO eigenvalue and ionization potential. This optimized ω value was employed in all LC‐ωPBE ground‐ and excited‐state calculations for the complete set of GQDs. Thus, a single fixed ω value was used consistently across the GQD dataset rather than individually optimizing ω for each structure.

The optimized ω value is significantly smaller than the default value of LC‐*ω*PBE (ω = 0.40 Bohr^−1^), which is consistent with extended *π*‐conjugated molecular systems. The optimal range‐separation parameter generally decreases as the extent of *π*‐electron delocalization increases because long‐range exchange interactions become effective over larger distances [40]. Consequently, smaller ω values are required to accurately reproduce frontier orbital energies, ionization potential, and excited‐state properties in large *π*‐conjugated systems.

## 3 Numerical Results

### 3.1 Semiempirical‐Predicted Size Band‐Gap Relationship

The simplest way to decrease the band gap and red‐shift the emission of a graphitic nanostructure is to increase the conjugation length and molecular size. However, our goal is to identify near‐IR chromophores present in relatively small 1–4 nm GQDs. Accordingly, we first confirm that the generated structures do not exhibit a trivial relationship between smaller gaps and larger sizes. Figure 3a shows the semiempirically predicted absorption wavelengths of 3000 structures with molecular weights between 800 and 1600 g/mol. The weak correlation between these properties demonstrates the chemical diversity of the generated structures, which is a prerequisite for identifying real chromophores. Figure 3b shows that the computed HOMO and LUMO energies are strongly correlated: molecules with a more negative HOMO have a more positive LUMO and, correspondingly, a larger band gap.

**FIGURE 3:**
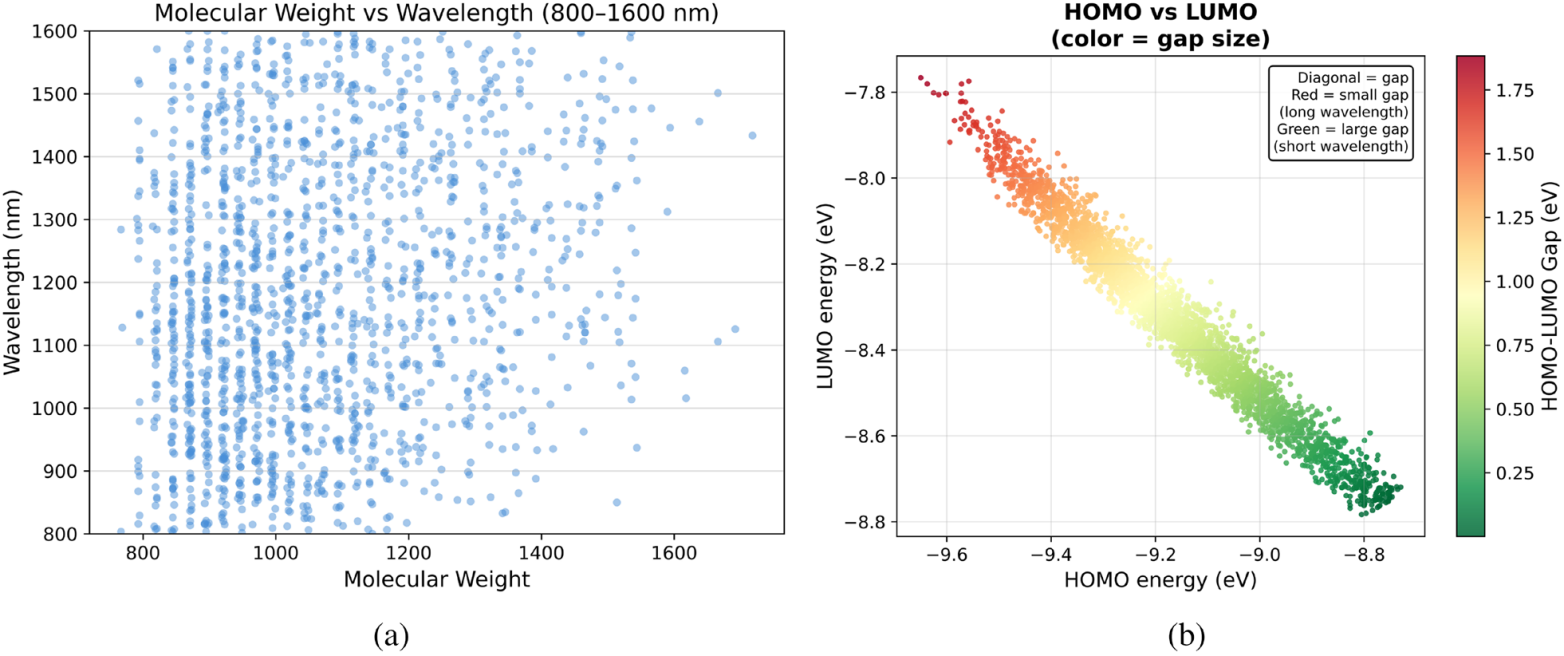
(a) Relationship between molecular weight and predicted absorption wavelength (700–1700 nm range) for the fusion‐based GQD structures. (b) Correlation between computed HOMO and LUMO energies, colored by HOMO–LUMO gap size; molecules with a more negative HOMO have a more positive LUMO and a larger band gap.

### 3.2 Machine Learning Model Performance for HOMO‐LUMO Gap Prediction

A key advance of the current work is the use of a machine learning model to rapidly predict the semiempirical GFN2‐xTB band gap from molecular features, including Morgan fingerprints, MACCS structural keys (a 167‐bit fingerprint encoding predefined chemical substructures), and RDKit‐derived two‐dimensional molecular descriptors. These features were computed directly from the two‐dimensional molecular structures without requiring three‐dimensional geometry optimization or an explicit GFN2‐xTB calculation. We carried out a comparison between multiple training protocols. Each model was trained on a dataset of 4000 structures and evaluated on a separate test set of 1000 structures.

We compared the performance of five machine learning models: Random Forest, XGBoost, LightGBM, CatBoost, and a Neural Network. Model performance on the test dataset was evaluated using the coefficient of determination ($R^{2}$) and root mean square error (RMSE). Among all the evaluated models, LightGBM showed the best predictive performance, with an $R^{2}$ of 0.6716 and an RMSE of 0.2297 eV, followed by CatBoost with $R^{2}=0.6439$ and RMSE = 0.2392 eV and XGBoost with $R^{2}=0.6380$ and RMSE = 0.2412 eV. Random Forest and the Neural Network showed lower predictive performance, with $R^{2}$ values of 0.5690 and 0.5428, respectively. Based on these results, LightGBM was selected to screen the full structure library and identify candidates with HOMO–LUMO gaps corresponding to the target NIR range. The predictive performance of the five machine learning models is summarized in Table 1.

**TABLE 1:** Performance comparison of machine learning models for HOMO‐LUMO gap prediction using a 4000‐molecule training set and a 1000‐molecule test set.

Model	MAE	RMSE	*R* ^2^
LightGBM	0.1839	0.2297	0.6716
CatBoost	0.1913	0.2392	0.6439
XGBoost	0.1948	0.2412	0.6380
Random forest	0.2118	0.2632	0.5690
Neural network	0.2160	0.2711	0.5428

### 3.3 SHAP Analysis of the LightGBM Model

We performed SHAP (Shapley Additive Explanations) analysis to interpret the features learned by the LightGBM model using the same scaled feature representation employed during model training on the 4000 molecule training set. As per the SHAP feature importance ranking, Morgan fingerprint was one of the important features predicting HOMO‐LUMO gap, as there were several Morgan features which had higher importance than RDKit two‐dimensional descriptors. The important feature was Morgan_R3_1680, with a mean absolute SHAP value of 0.0748 eV. This feature was found in 2574(64.4%) out of the total 4000 training structures. In all 2574 structures containing this feature, the SHAP value was negative, with mean value of −0.0611 eV and a median value of −0.0588 eV. This indicates that this particular substructure played an important role in predicting a smaller HOMO‐LUMO gap in the LightGBM model. The structural pattern of Morgan_R3_1680 was understood through the decoding of the fingerprint and the local substructure SMILES ccc1ccccc1c, which is part of an extended aromatic system that includes a benzene‐ring structure. The complete top 20 features SHAP summary plot and feature‐importance ranking are provided in Figure 4 and Table 2.

**FIGURE 4:**
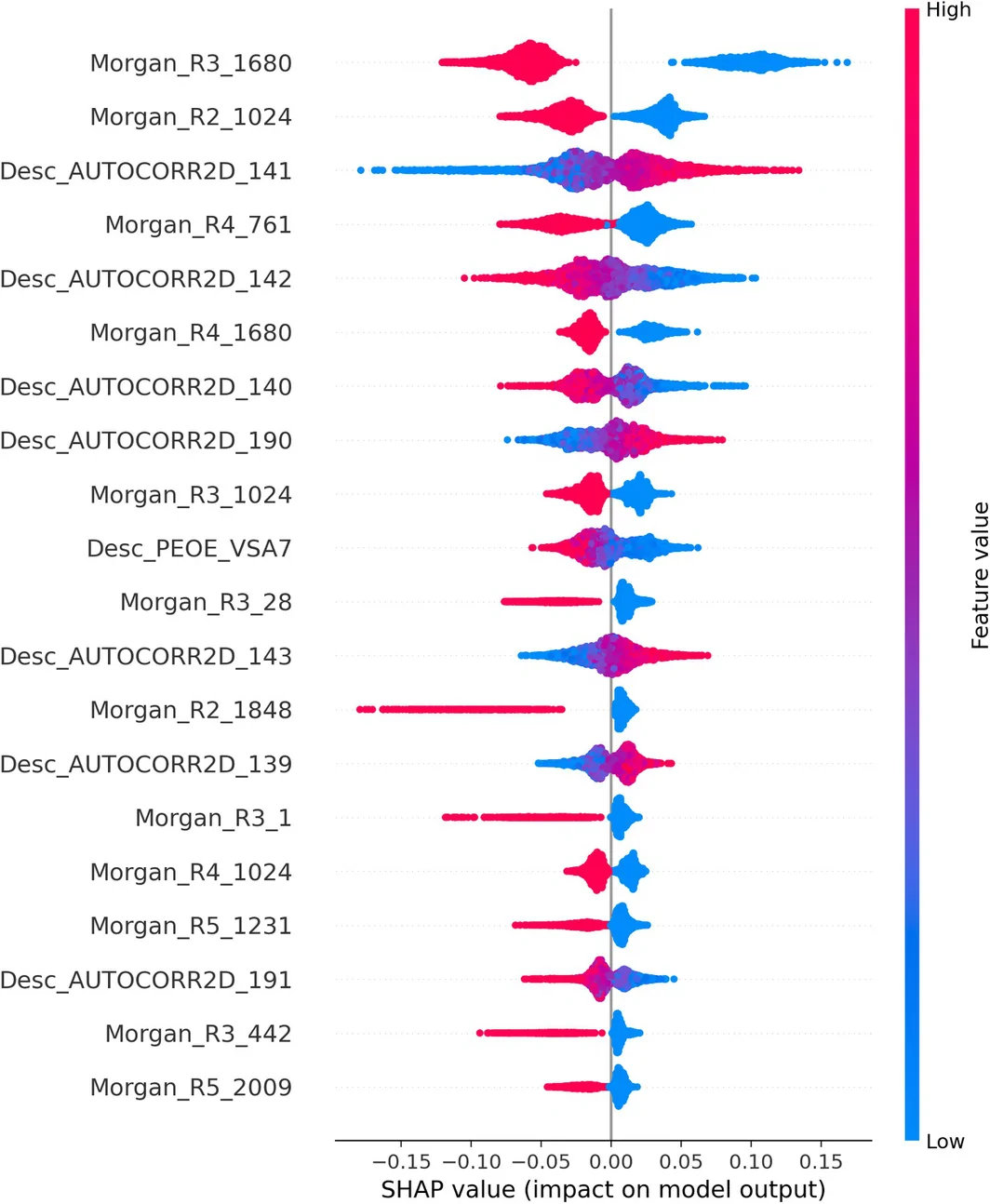
SHAP summary plot for the LightGBM model, showing the top 20 features from the training set. Each point is one molecule, colored by that feature's value (red = high, blue = low), with position showing its effect on the predicted HOMO–LUMO gap.

**TABLE 2:** Top 20 features ranked by mean absolute SHAP value for the LightGBM model, based on the training set.

Rank	Feature	Mean SHAP
1	Morgan_R3_1680	0.0748
2	Morgan_R2_1024	0.0347
3	Desc_AUTOCORR2D_141	0.0319
4	Morgan_R4_761	0.0281
5	Desc_AUTOCORR2D_142	0.0258
6	Morgan_R4_1680	0.0212
7	Desc_AUTOCORR2D_140	0.0192
8	Desc_AUTOCORR2D_190	0.0184
9	Morgan_R3_1024	0.0175
10	Desc_PEOE_VSA7	0.0165
11	Morgan_R3_28	0.0161
12	Desc_AUTOCORR2D_143	0.0146
13	Morgan_R2_1848	0.0137
14	Desc_AUTOCORR2D_139	0.0126
15	Morgan_R3_1	0.0123
16	Morgan_R4_1024	0.0122
17	Morgan_R5_1231	0.0113
18	Desc_AUTOCORR2D_191	0.0110
19	Morgan_R3_442	0.0101
20	Morgan_R5_2009	0.0099

### 3.4 Top 50 NIR‐Active GQD Candidates

The semiempirical and TD‐DFT results for the 50 selected structures are listed in Table 3. The selected structures have similar GFN2‐xTB HOMO–LUMO gaps corresponding to the target NIR range. According to TD‐DFT calculations, all structures are predicted to exhibit NIR emission. The molecular weights range from approximately 820 to 1458 g/mol, while the molecular diameters range from 1.83–2.96 nm with a mean diameter of 2.33 nm, depending on the number of aromatic carbon atoms in the graphene‐like core.

**TABLE 3:** Molecular formulas, Molecular weights (MW), GFN2‐xTB HOMO–LUMO gaps, and corresponding GFN2‐xTB‐calculated absorption wavelengths, along with TD‐DFT‐computed excitation ($S_{0}\to S_{1}$) and fluorescence ($S_{1}\to S_{0}$) wavelengths obtained using the long‐range‐corrected LC‐*ω*PBE (ω = 0.15 Bohr^−1^) functional with the def2‐SVP basis set in the gas phase.

Quantum dots	Molecular formula	MW (g/mol)	xTB Gap (eV)	xTB λabsorption (nm)	LC‐ωPBE excitation (nm)	LC‐ωPBE fluorescence (nm)	Heat of formation (KJ/mol)	Heat of formation (KJ/g)	SA score
1	C_72_H_32_	897.05	0.7342	1689	794	883	837	0.93	3.45
2	C_70_H_30_	871.01	0.7348	1688	736	806	823	0.94	3.31
3	C_70_H_28_	868.99	0.7344	1688	809	886	757	0.87	3.47
4	C_82_H_34_	1019.17	0.7341	1689	815	903	952	0.93	3.61
5	C_68_H_30_	846.99	0.7353	1686	721	780	872	1.03	3.30
6	C_76_H_34_	947.11	0.7353	1686	765	858	1041	1.10	3.47
7	C_86_H_42_	1075.28	0.7344	1688	725	816	1256	1.17	3.81
8	C_80_H_34_	995.15	0.7354	1686	830	1139	1020	1.03	3.56
9	C_92_H_40_	1145.33	0.7339	1690	749	814	1193	1.04	3.99
10	C_74_H_32_	921.07	0.7361	1684	811	914	913	0.99	3.40
11	C_82_H_36_	1021.19	0.7349	1687	810	965	1011	0.99	3.78
12	C_88_H_34_	1091.24	0.7345	1688	783	854	929	0.85	3.92
13	C_70_H_32_	873.03	0.7377	1681	760	836	959	1.10	3.20
14	C_68_H_30_	846.99	0.7376	1681	748	844	857	1.01	3.26
15	C_76_H_30_	943.08	0.7363	1684	785	861	789	0.84	3.63
16	C_80_H_36_	997.17	0.7362	1684	745	837	1009	1.01	3.65
17	C_74_H_32_	921.07	0.7365	1684	843	1070	846	0.92	3.59
18	C_86_H_38_	1071.25	0.7359	1685	808	928	1096	1.02	3.77
19	C_78_H_34_	971.13	0.7362	1684	796	873	920	0.95	3.69
20	C_82_H_36_	1021.19	0.7363	1684	815	909	1087	1.06	3.69
21	C_90_H_38_	1119.29	0.7350	1687	781	868	1022	0.91	4.06
22	C_76_H_36_	949.12	0.7376	1681	846	992	1131	1.19	3.41
23	C_80_H_34_	995.15	0.7379	1680	784	860	992	1.00	3.70
24	C_112_H_38_	1383.54	0.7338	1690	805	881	1103	0.80	4.83
25	C_86_H_40_	1073.27	0.7375	1681	835	1064	1124	1.05	3.80
26	C_76_H_32_	945.09	0.7385	1679	770	829	883	0.93	3.62
27	C_78_H_34_	971.13	0.7396	1677	818	931	1019	1.05	3.33
28	C_88_H_42_	1099.30	0.7380	1680	806	906	1284	1.17	3.89
29	C_72_H_34_	899.06	0.7403	1675	821	953	944	1.05	3.37
30	C_98_H_34_	1211.35	0.7368	1683	812	878	984	0.81	4.35
31	C_66_H_28_	820.95	0.7414	1672	750	880	826	1.01	3.18
32	C_94_H_36_	1165.32	0.7382	1680	814	902	1016	0.87	4.09
33	C_66_H_28_	820.95	0.7416	1672	786	868	820	1.00	3.25
34	C_98_H_42_	1219.41	0.7378	1681	756	846	1245	1.02	4.31
35	C_78_H_36_	973.15	0.7410	1674	813	948	945	0.97	3.48
36	C_68_H_30_	846.99	0.7417	1672	814	919	832	0.98	3.30
37	C_78_H_34_	971.13	0.7406	1674	738	795	903	0.93	3.64
38	C_110_H_46_	1367.58	0.7367	1683	797	874	1312	0.96	4.71
39	C_76_H_34_	947.11	0.7415	1672	771	847	1044	1.10	3.46
40	C_74_H_30_	919.05	0.7414	1672	765	833	832	0.91	3.54
41	C_118_H_40_	1457.62	0.7362	1684	795	926	1217	0.84	5.03
42	C_76_H_34_	947.11	0.7424	1670	797	912	919	0.97	3.34
43	C_110_H_46_	1367.58	0.7375	1681	812	947	1260	0.92	4.68
44	C_104_H_36_	1285.43	0.7381	1680	813	877	1020	0.79	4.55
45	C_80_H_38_	999.18	0.7415	1672	774	913	1030	1.03	3.62
46	C_90_H_44_	1125.34	0.7406	1674	837	975	1299	1.15	3.87
47	C_82_H_36_	1021.19	0.7413	1673	776	914	1034	1.01	3.77
48	C_116_H_40_	1433.60	0.7371	1682	779	849	1229	0.86	4.96
49	C_80_H_36_	997.17	0.7420	1671	803	917	1042	1.05	3.69
50	C_70_H_30_	871.01	0.7431	1669	701	777	835	0.96	3.44

*Note:* The corresponding heats of formation ($\Delta H_{f}$) were calculated at the B3LYP/def2‐SVP level of theory, and synthetic accessibility (SA) scores were computed using RDKit.

#### 3.4.1 Excitation and Fluorescence Properties

The top 50 GQD structures selected for TD‐DFT calculations based on their low GFN2‐xTB computed band gaps and synthetic accessibility scores are shown in Figure S1, and the ground‐state optimized geometries are presented in Figure S2. All selected candidates exhibit excitation and fluorescence wavelengths within the NIR window. The red‐shifted fluorescence is characterized by a highly extended *π*‐conjugated framework and a non‐compact, elongated molecular architecture with multiple peripheral *π*‐conjugated extensions. The combination of an extended *π*‐conjugated framework, increased effective conjugation length, and elongated molecular topology enhances *π*‐electron delocalization, resulting in a reduced HOMO‐LUMO energy gap. Consequently, the lowest singlet excited state is stabilized, leading to a lower‐energy $S_{1}\to S_{0}$ transition. The frontier molecular orbitals (FMOs) of representative GQDs involved in the lowest‐energy electronic excitation are shown in Figure 5. Both the HOMO and LUMO are extensively delocalized over the same fused *π*‐conjugated framework, indicating that these regions collectively constitute the optically active chromophore responsible for NIR excitation. The significant overlap between the HOMO and LUMO suggests that the lowest‐energy excitation is predominantly a delocalized $\pi \to \pi^{*}$ transition. TD‐DFT excitation analysis also shows that $S_{0}\to S_{1}$ excitation is dominated by the HOMO → LUMO transition, while the fluorescence emission primarily involves the relaxation between the same pair of frontier orbitals. The similar spatial distribution of the HOMO and LUMO across the representative GQD structures indicates that the irregular extended *π*‐conjugated framework is primarily responsible for both the NIR excitation and fluorescence throughout the investigated series.

**FIGURE 5:**
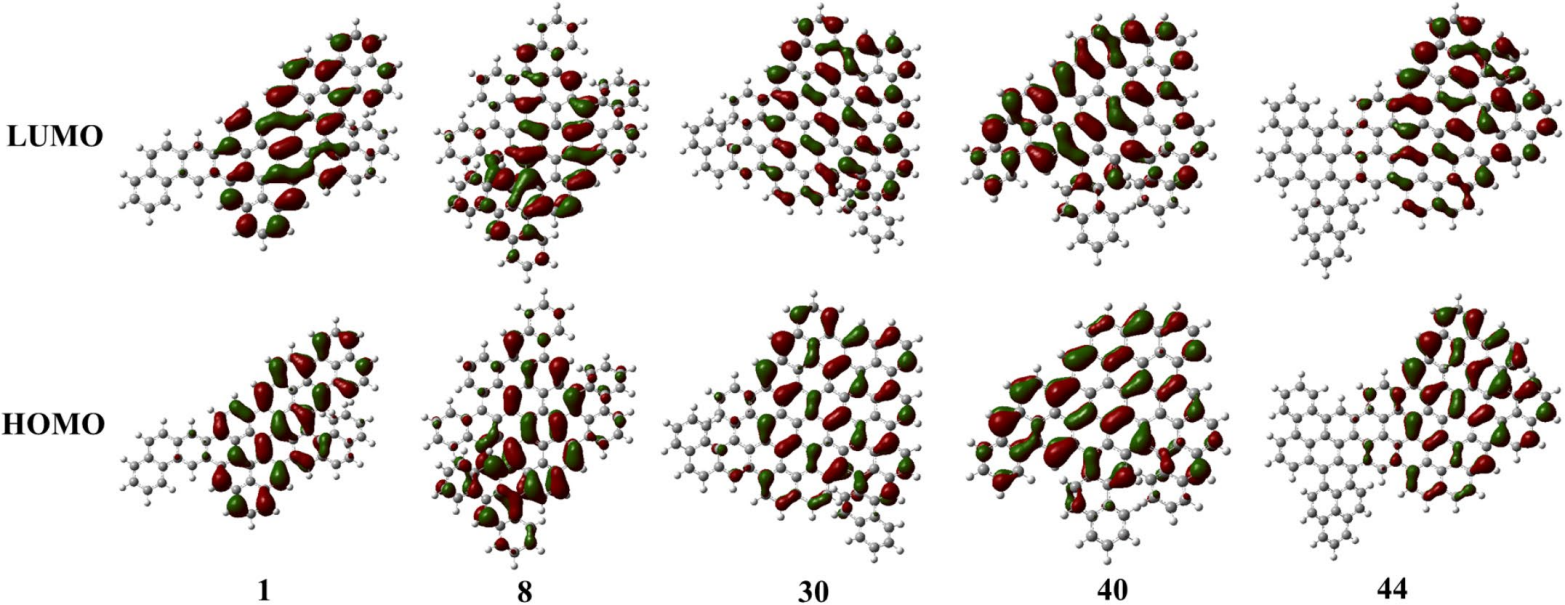
Frontier molecular orbitals (HOMO and LUMO) of representative GQDs illustrating the delocalized π‐conjugated chromophore responsible for the lowest‐energy $\pi \to \pi^{*}$ excitation and fluorescence.

In our previous study, we hypothesized that several electronic and structural factors contribute to the NIR emission, including extended π‐conjugation, charge‐transfer excitations, diradicaloid character, stacking of multiple GQD layers, and blocking of nonradiative decay pathways (as seen in nonaromatic fluorescence) [18]. These compounds do not exhibit open‐shell singlet diradical character, as evidenced by the absence of spin contamination ($\left\langle S^{2}\right\rangle =0$) in the broken‐symmetry calculations. The low‐lying excited states are optically forbidden ($f_{0}=0$) within the broken‐symmetry formalism. The excitation wavelength of the first optically allowed excited state is essentially the same as predicted by spin‐restricted TD‐DFT calculations. The TD‐DFT‐Computed excitation and fluorescence wavelengths, along with the heats of formation ($\Delta H_{f}$) and synthetic accessibility (SA) scores, are summarized in Table 3. The B3LYP/def2‐SVP computed fluorescence emission wavelengths are provided in Table S1.

#### 3.4.2 Synthetic Accessibility and Thermodynamic Stability

The correlation and scatter plots between heats of formation ($\Delta H_{f}$) and synthetic accessibility (SA) scores of the 50 GQDs selected for TD‐DFT calculations are shown in Figure 6. The SA score [19] was employed as an initial screening descriptor to identify synthetically accessible candidates from the large GQD library, while the heat of formation was calculated to evaluate their thermodynamic stability. A moderate positive correlation (R^2^ = 0.441) between $\Delta H_{f}$ and SA score indicates that the larger and more structurally complex GQDs generally have higher formation energies and higher SA scores, suggesting greater synthetic difficulty. However, the scatter in the data shows that synthetic accessibility and thermodynamic stability are complementary descriptors, with each providing independent information for prioritizing candidate GQDs. Although neither descriptor describes the complete synthetic pathway or experimental feasibility, their combined use provides a practical and computationally efficient strategy for identifying promising GQDs that balance thermodynamic stability and synthetic accessibility.

**FIGURE 6:**
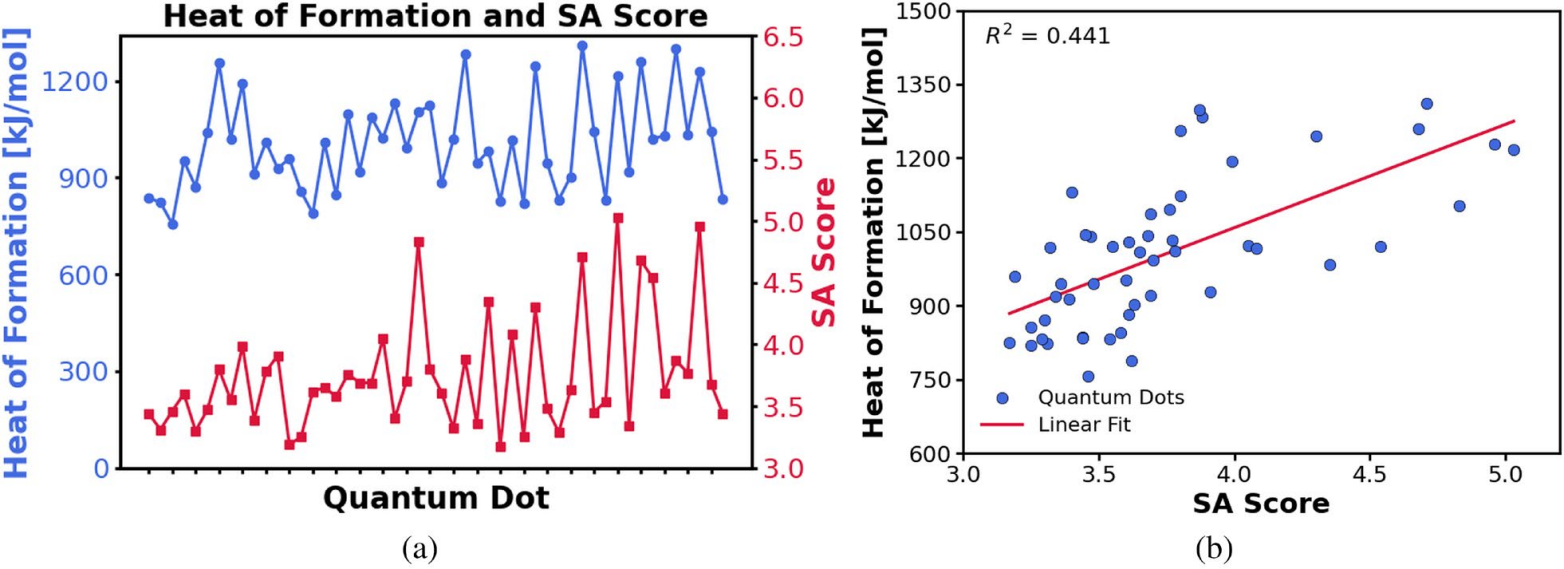
Correlation between heat of formation and SA score computed using ground‐state B3LYP‐optimized energies and RDKit, respectively.

## 4 Conclusion

This study presents a computational workflow for generating and screening fusion‐based GQDs with near‐infrared (NIR) optical properties. A fusion‐based hexagonal lattice growth approach was used to generate 100,000 candidate graphitic structures. Molecular descriptors and synthetic accessibility (SA) scores were calculated for the generated structures to provide initial measures of structural properties and synthetic accessibility. GFN2‐xTB band gap calculations were performed for a randomly selected subset of 5000 structures and used to train and evaluate five machine learning models (Random Forest, XGBoost, LightGBM, CatBoost, and Neural Network). Among these models, LightGBM showed the best predictive performance and was used to predict the band gaps of the remaining 95,000 structures.

ML‐predicted band gaps were used to pre‐screen 11,000 structures with low predicted band gaps, followed by GFN2‐xTB calculations. Based on these calculations, the top 50 candidates with low SA score and low GFN2‐xTB band gap were selected for TD‐DFT calculations using both B3LYP and long‐range‐corrected LC‐ωPBE functionals. The 50 selected candidates were predicted by TD‐DFT to exhibit emission within the NIR spectral region. Overall, this computational framework demonstrates that the combination of rule‐based molecular generation, semiempirical quantum chemistry calculations, machine learning, and time‐dependent density functional theory (TD‐DFT) can efficiently explore large GQD chemical spaces, providing a computational database of candidate NIR‐emitting GQDs for future experimental validation.

## Funding

This work was supported by Welch Foundation (X‐P‐0012‐20250717).

## Conflicts of Interest

The authors declare no conflicts of interest.

## Supporting information


**Figure S1:** Top 50 candidate graphitic structures selected based on low GFN2‐xTB‐calculated band gaps and low SA scores for subsequent TD‐DFT calculations. TD‐DFT calculations predict that these structures exhibit near‐infrared (NIR) emission.
**Table S1:** B3LYP/def2‐SVP computed fluorescence emission wavelengths corresponding to the S_1_ → S_0_ transition for the investigated graphitic nanostructures
**Figure S2:** Ground‐state optimized geometries of the top 50 NIR‐emissive graphitic structures calculated at the B3LYP/def2‐SVP level of theory.

## Data Availability

The source code and scripts used in this study are available at https://github.com/nagamanigajula/GQD_Project_GH/tree/main.
