# Supplementary material for: Physics‐Based AI‐Augmented Workflows Discovering Candidate Graphene Quantum Dot Near‐Infrared Chromophores
Source: J Comput Chem. 2026 Nov 2;47(29):e70522. doi: 10.1002/jcc.70522 (PMC13726809; doi:10.1002/jcc.70522)
Supplement: Supplementary file 1 [file JCC-47-0-s001.pdf]

Supporting Information

Physics-Based AI-Augmented Workflows Discovering Candidate Graphene Quantum Dot Near-Infrared Chromophores

Naga Mani Gajula[1‡], Muhammad Hammad Ali[2‡], Md. Masud Rana[1*], and Benjamin G. Janesko[2*]

[1]Department of Computer Science, Lamar University, Beaumont, TX 77705, USA

[2]Department of Chemistry & Biochemistry, Texas Christian University, Fort Worth, TX 76129, USA

***The Corresponding Author** (mrana15@lamar.edu and b.janesko@tcu.edu)

Author Contributions ‡These authors contributed equally.

1. Top 50 Candidate Graphitic Structures:

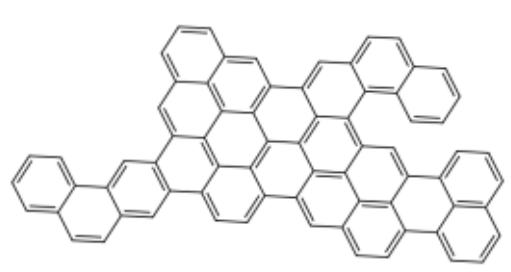

1. $C_{72}H_{32}$

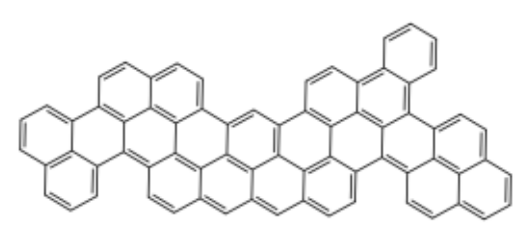

2. $C_{70}H_{30}$

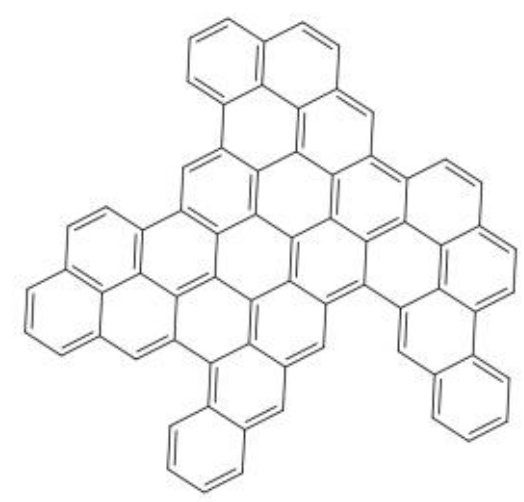

3. $C_{70}H_{28}$

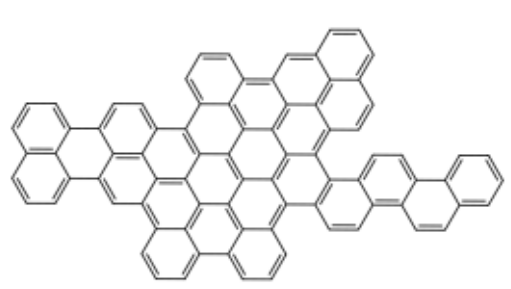

4. $C_{82}H_{34}$

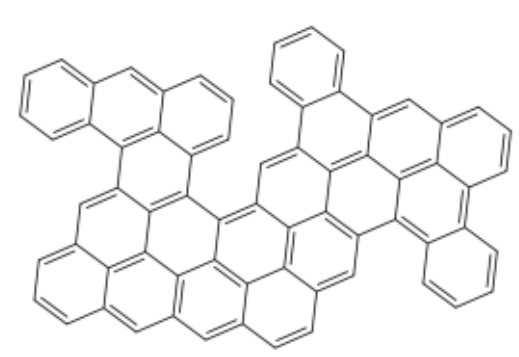

5. $C_{68}H_{30}$

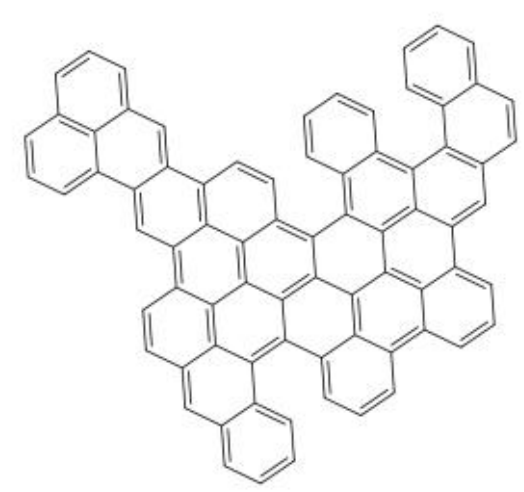

6. $C_{76}H_{34}$

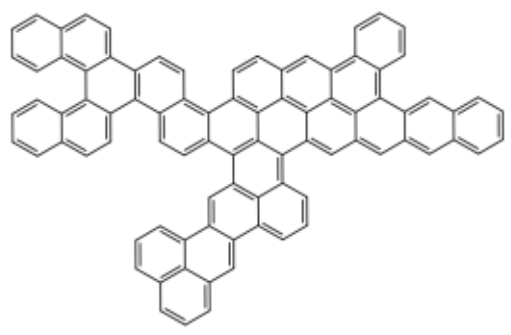

7. $C_{86}H_{42}$

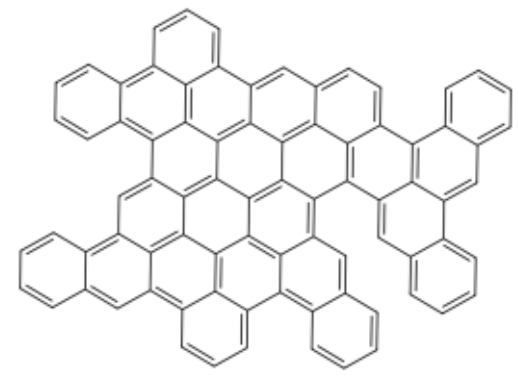

8. $C_{80}H_{34}$

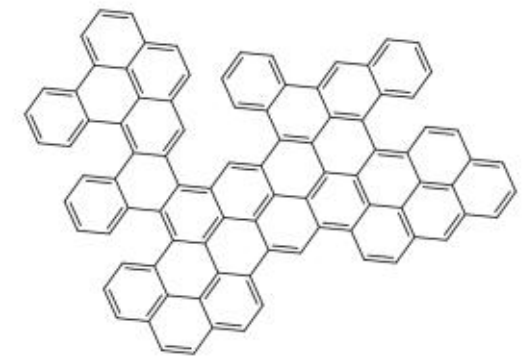

9. $C_{92}H_{40}$

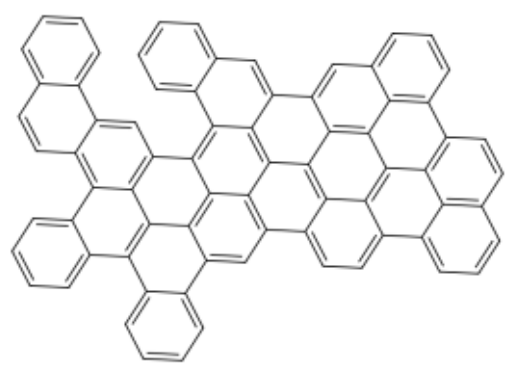

10. $C_{74}H_{32}$

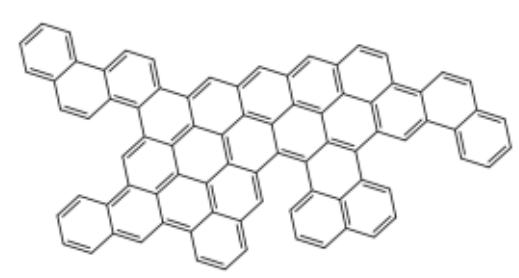

11. $C_{82}H_{36}$

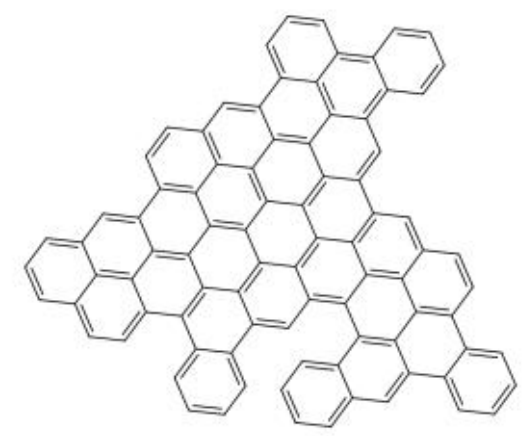

12. $C_{88}H_{34}$

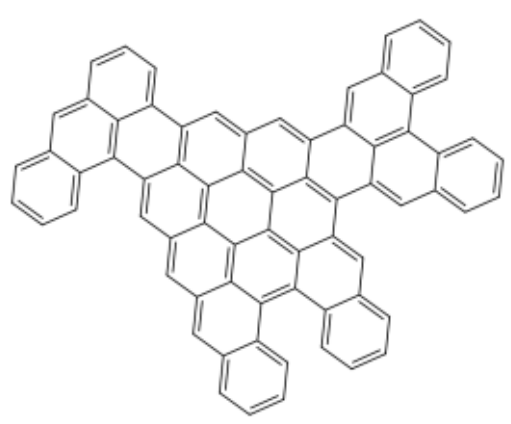

13. $C_{70}H_{32}$

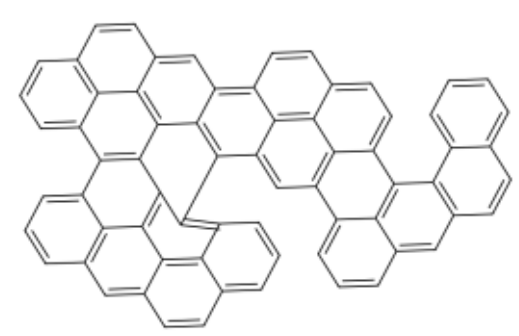

14. $C_{68}H_{30}$

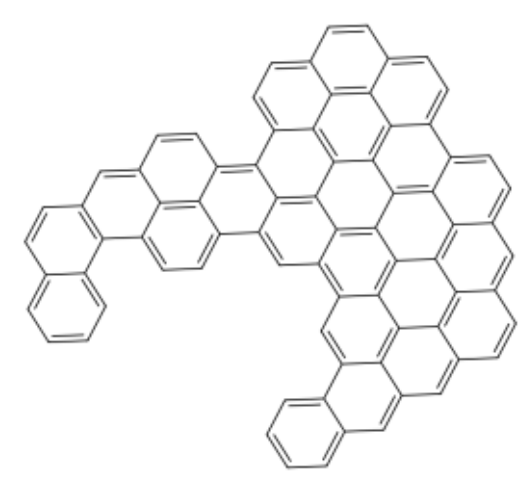

15. $C_{76}H_{30}$

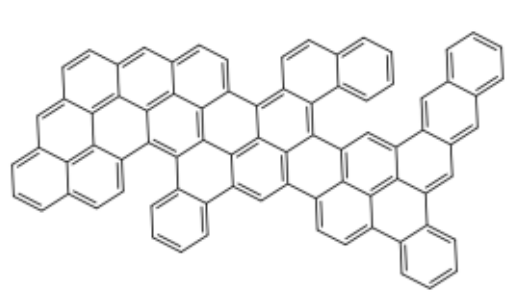

16. $C_{80}H_{36}$

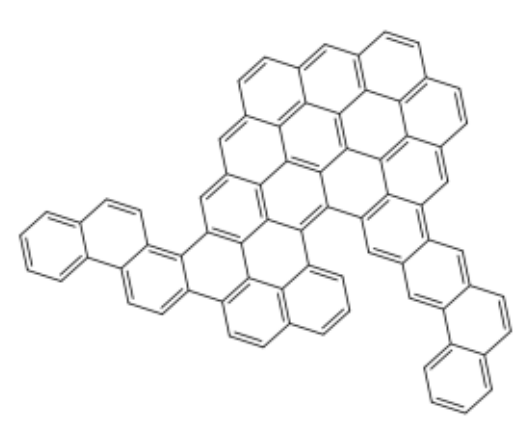

17. $C_{74}H_{32}$

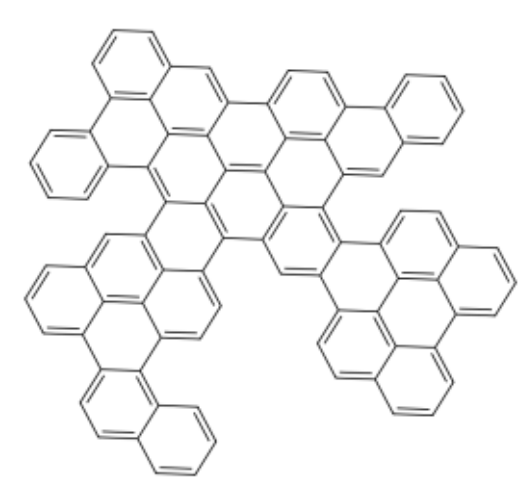

18. $C_{86}H_{38}$

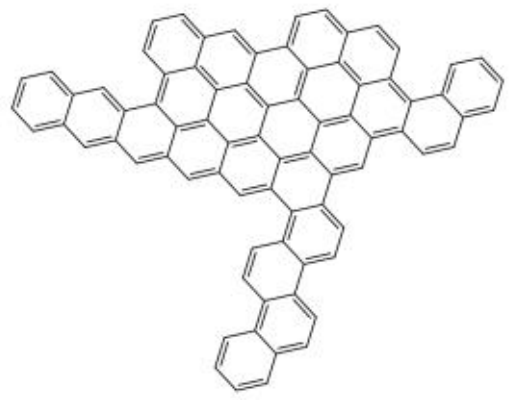

19. $C_{78}H_{34}$

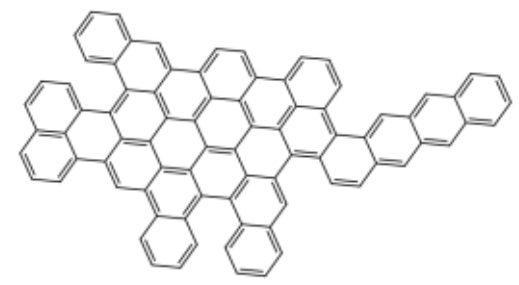

20. $C_{82}H_{36}$

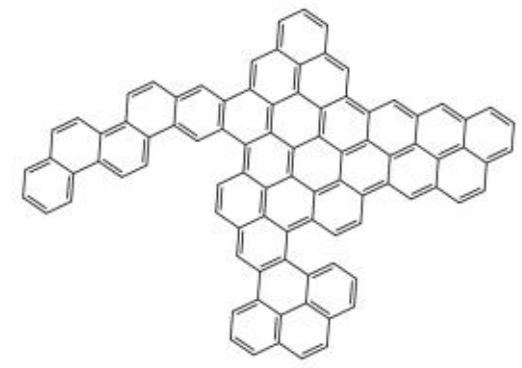

21. $C_{90}H_{38}$

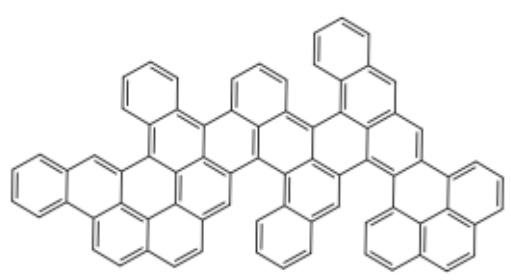

22. $C_{76}H_{36}$

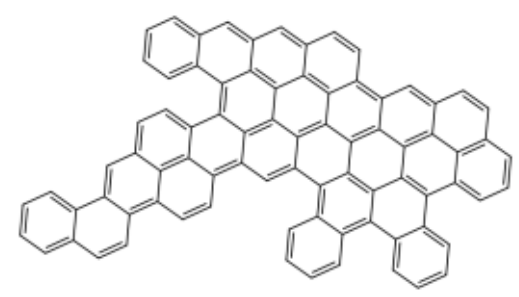

23. $C_{80}H_{34}$

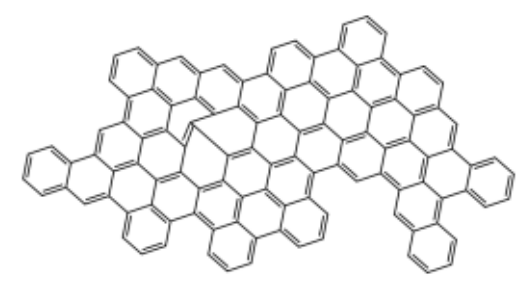

24. $C_{112}H_{38}$

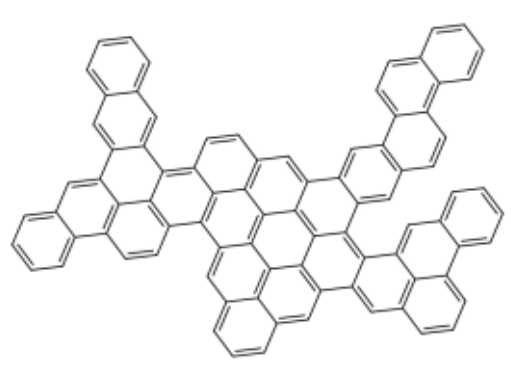

25. $C_{86}H_{40}$

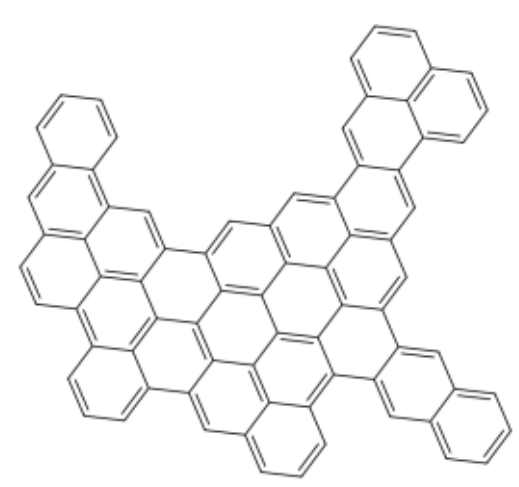

26. $C_{76}H_{32}$

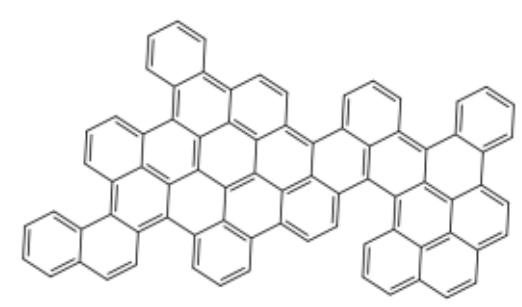

27. $C_{78}H_{34}$

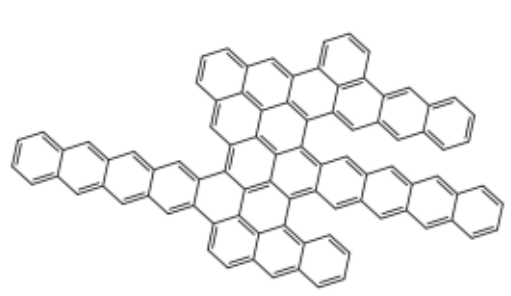

28. $C_{88}H_{42}$

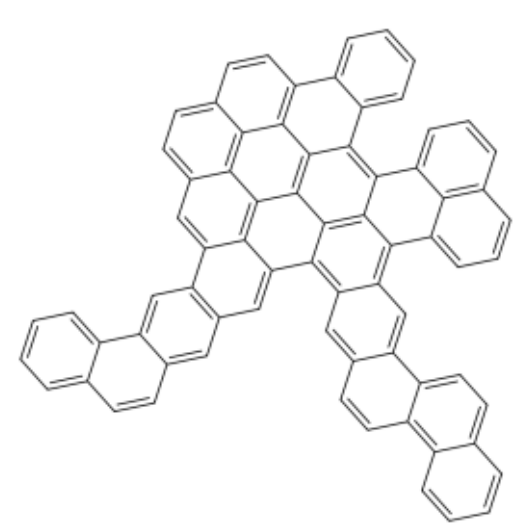

29. $C_{72}H_{34}$

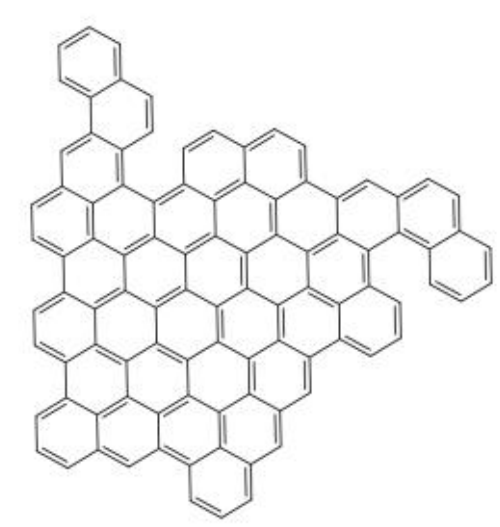

30. $C_{98}H_{34}$

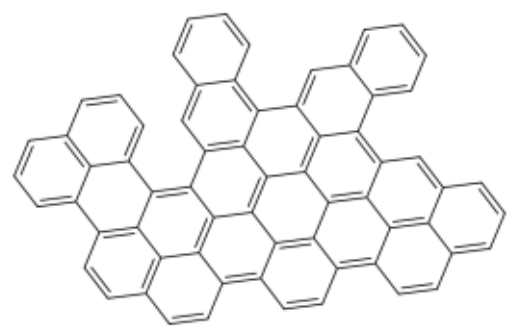

31. $C_{66}H_{28}$

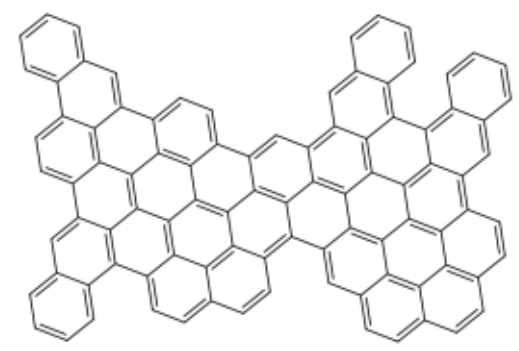

32. $C_{94}H_{36}$

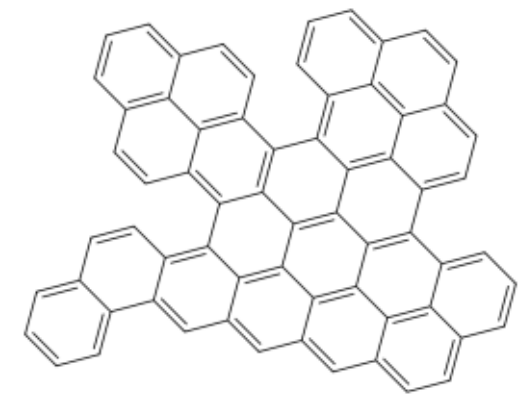

33. $C_{66}H_{28}$

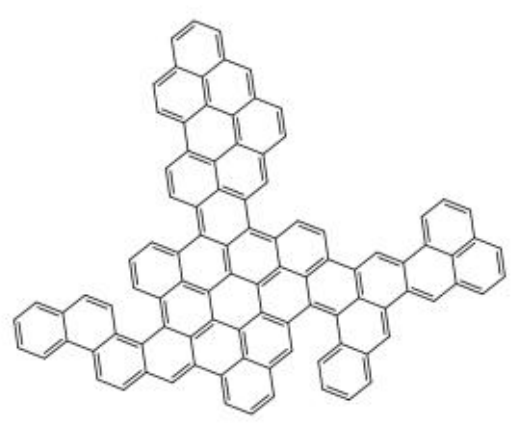

34. $C_{98}H_{42}$

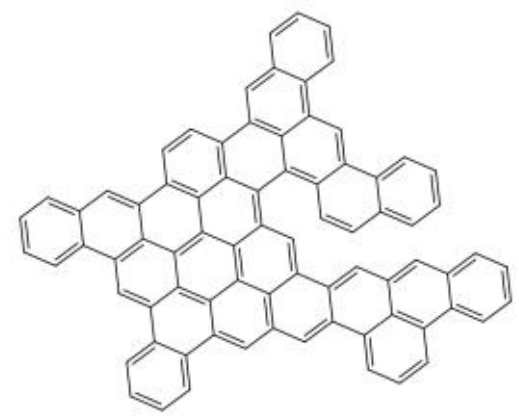

35. $C_{78}H_{36}$

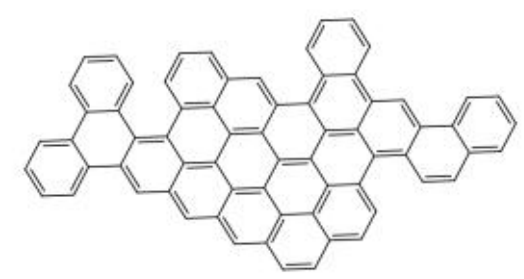

36. $C_{68}H_{30}$

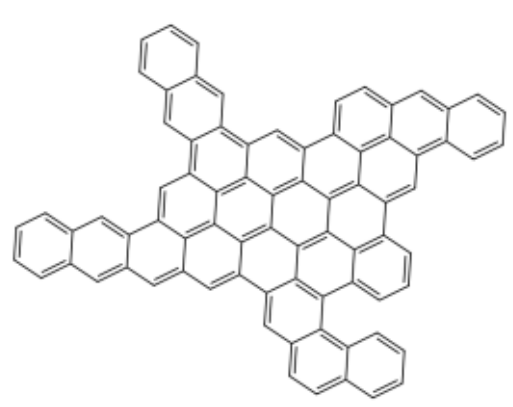

37. $C_{78}H_{34}$

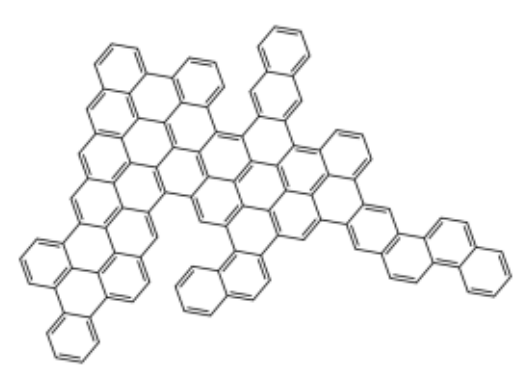

38. $C_{110}H_{46}$

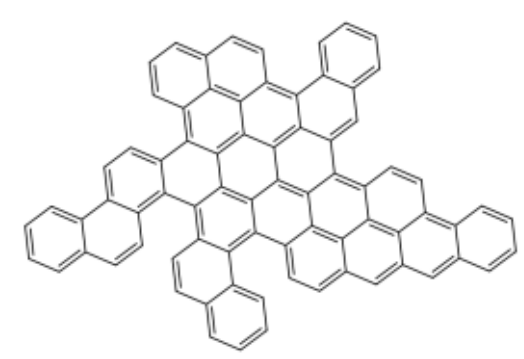

39. $C_{76}H_{34}$

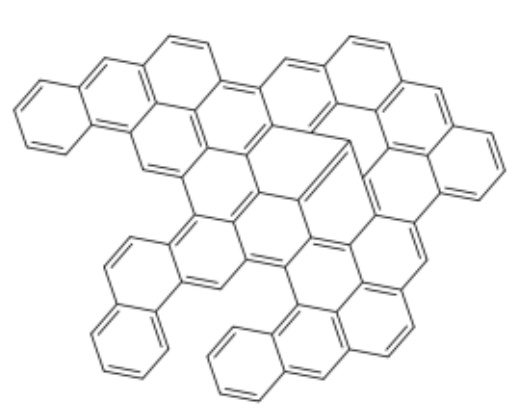

40. $C_{74}H_{30}$

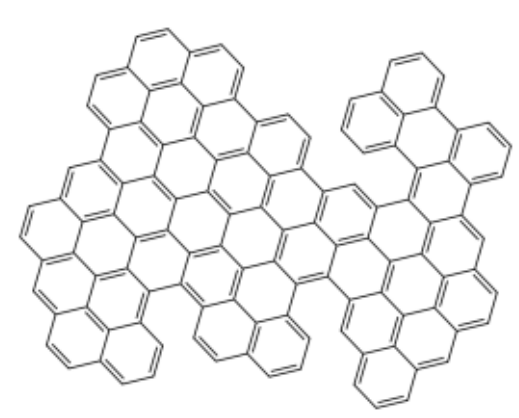

41. $C_{118}H_{40}$

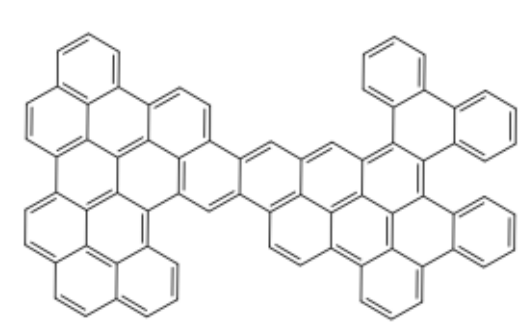

42. $C_{76}H_{34}$

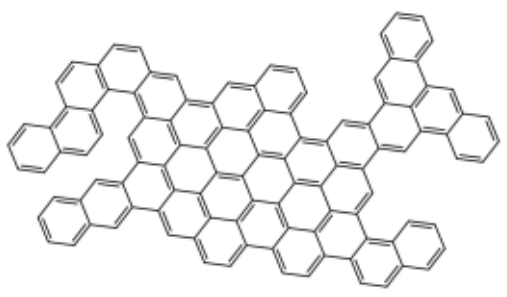

43. $C_{110}H_{46}$

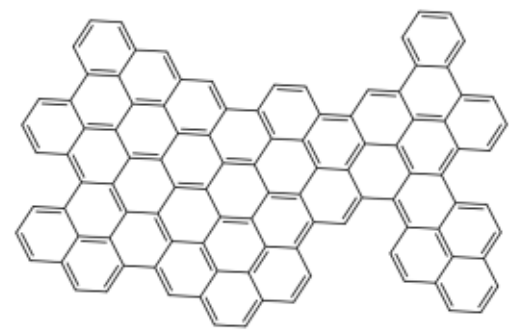

44. $C_{104}H_{36}$

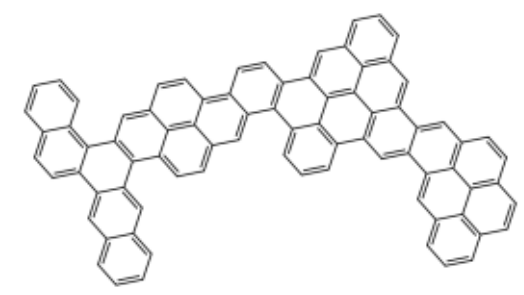

45. $C_{80}H_{38}$

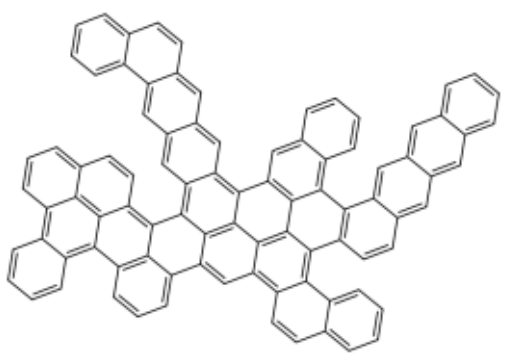

46. $C_{90}H_{44}$

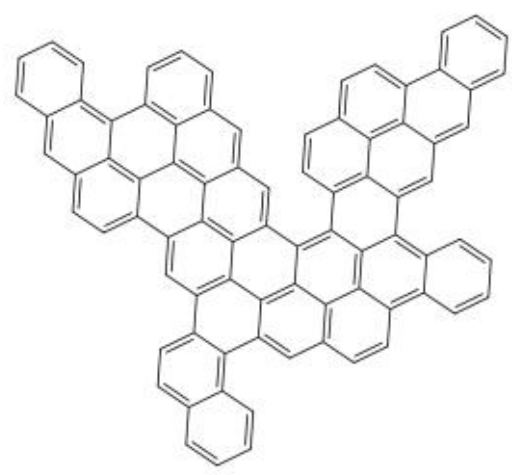

47. $C_{82}H_{36}$

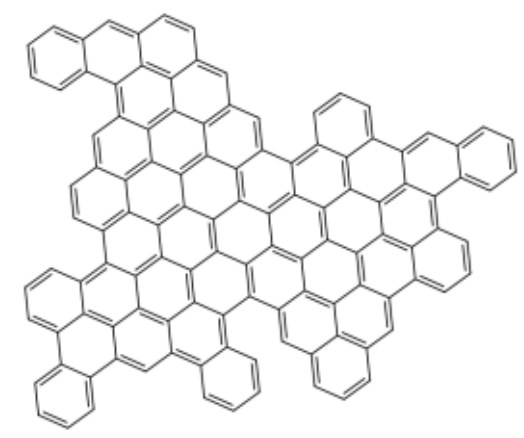

48. $C_{116}H_{40}$

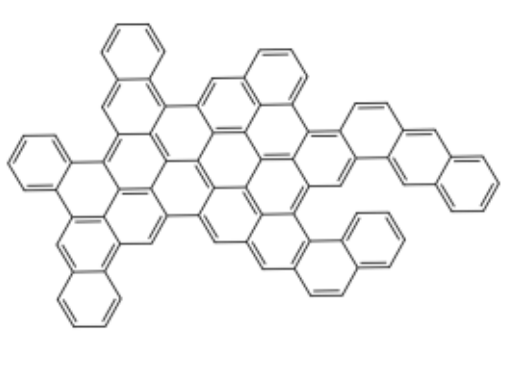

49. $C_{80}H_{36}$

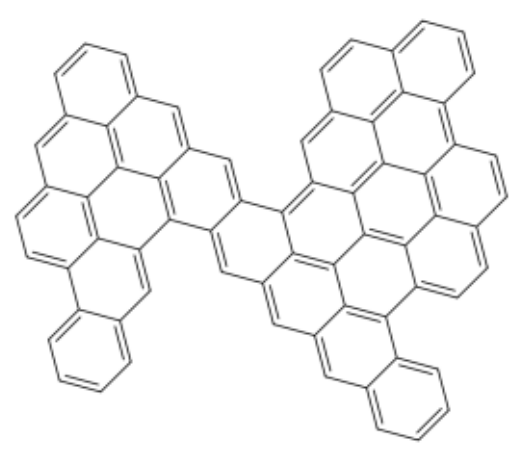

50. $C_{70}H_{30}$

Figure S1: Top 50 candidate graphitic structures selected based on low GFN2-xTB-calculated band gaps and low SA scores for subsequent TD-DFT calculations. TD-DFT calculations predict that these structures exhibit near-infrared (NIR) emission.

2. B3LYP/def2-SVP Computed Fluorescence Emission ($S_1 \rightarrow S_0$) Wavelengths:

Table S1: B3LYP/def2-SVP computed fluorescence emission wavelengths corresponding to the $S_1 \rightarrow S_0$ transition for the investigated graphitic nanostructures

Quantum Dots	λ (nm) Fluorescence (B3LYP)	Quantum Dots	λ (nm) Fluorescence (B3LYP)
**1**	914	**26**	983
**2**	837	**27**	980
**3**	970	**28**	1128
**4**	966	**29**	1020
**5**	894	**30**	986
**6**	876	**31**	897
**7**	901	**32**	978
**8**	1196	**33**	935
**9**	849	**34**	1004
**10**	960	**35**	1029
**11**	1037	**36**	1011
**12**	900	**37**	835
**13**	903	**38**	967
**14**	881	**39**	915
**15**	910	**40**	873
**16**	896	**41**	1029
**17**	1136	**42**	925
**18**	1045	**43**	971
**19**	978	**44**	968
**20**	974	**45**	925
**21**	929	**46**	1060
**22**	1069	**47**	964
**23**	944	**48**	907
**24**	952	**49**	988
**25**	1117	**50**	829

3. Ground-state Optimized Geometries:

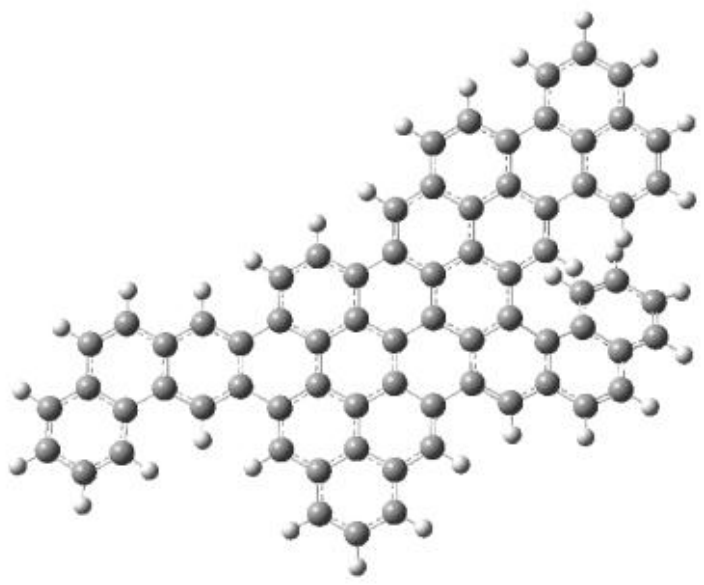

1

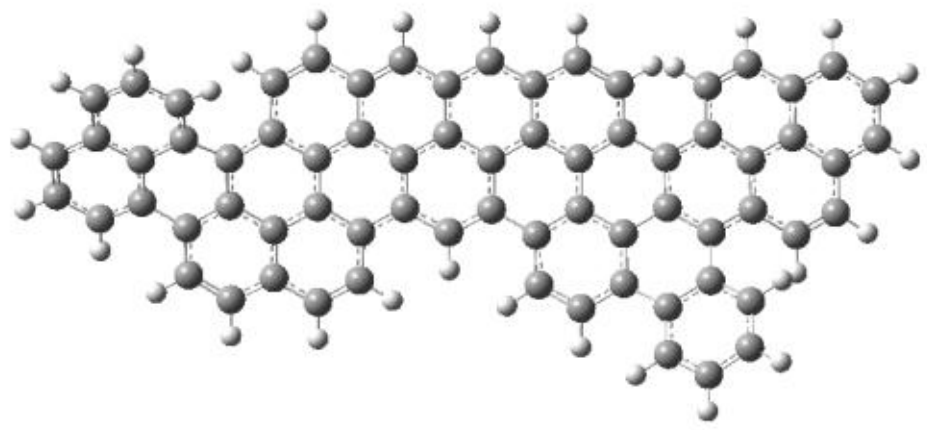

2

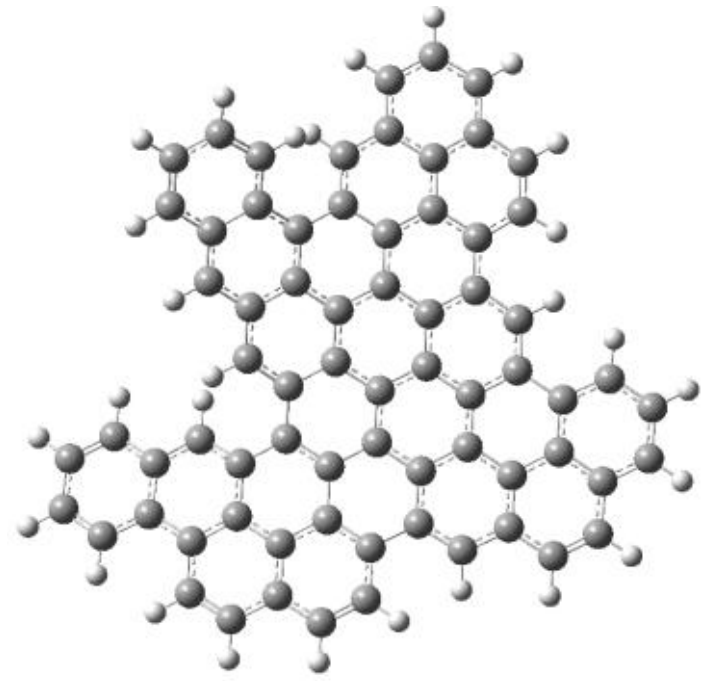

3

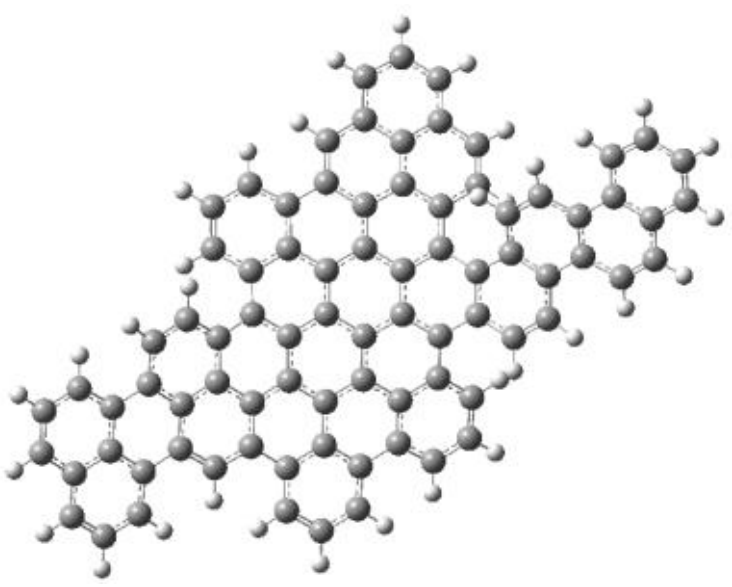

4

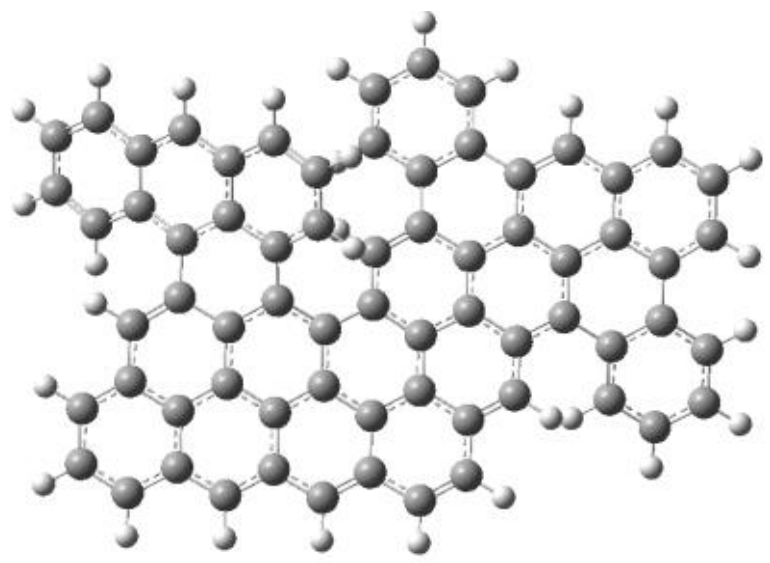

5

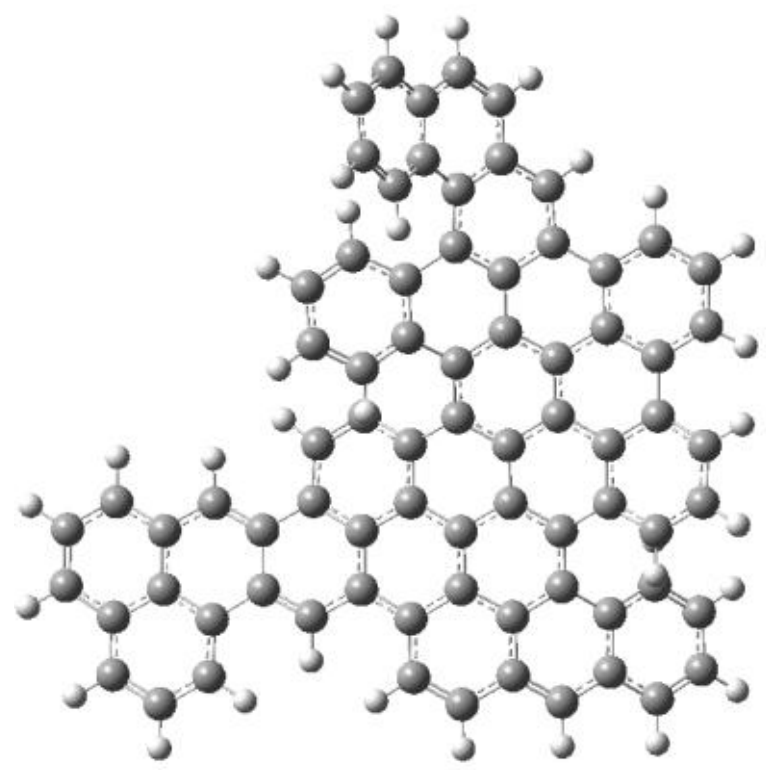

6

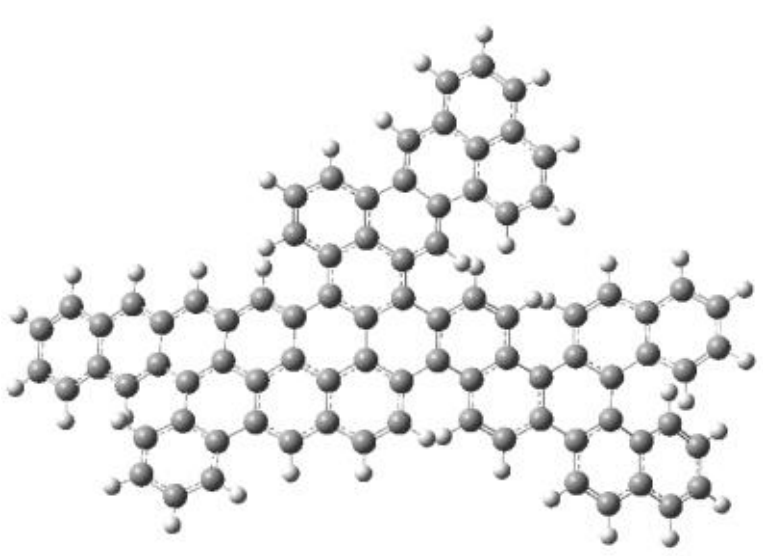

7

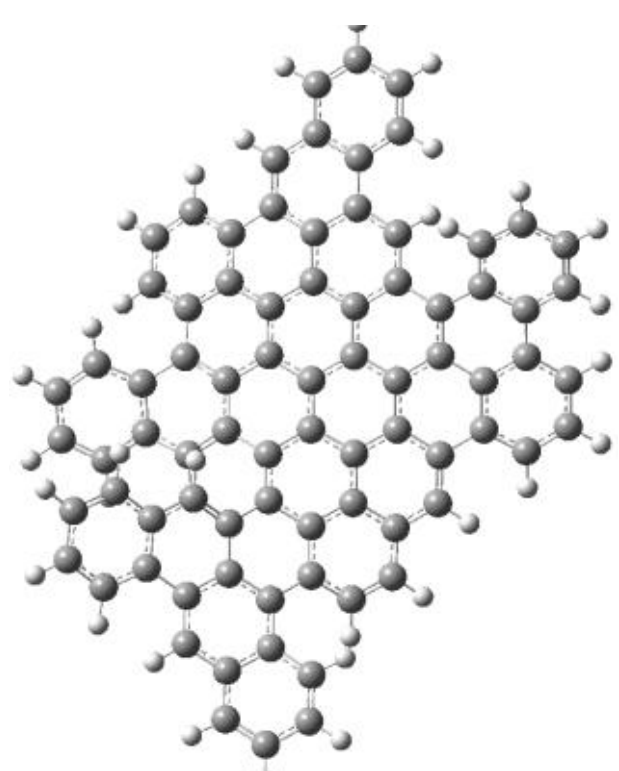

8

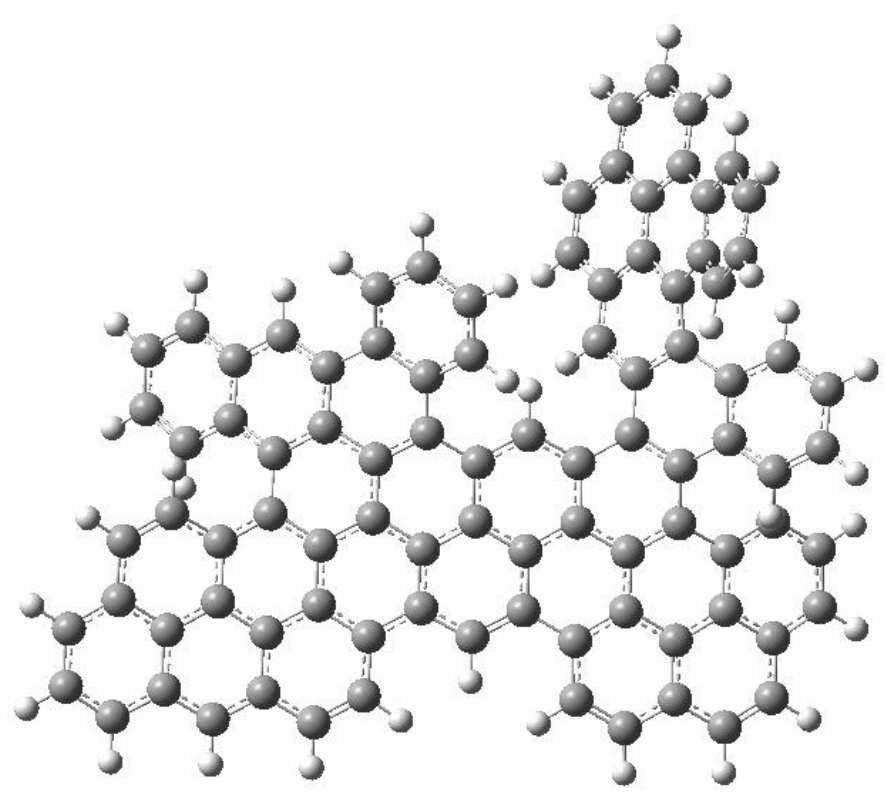

9

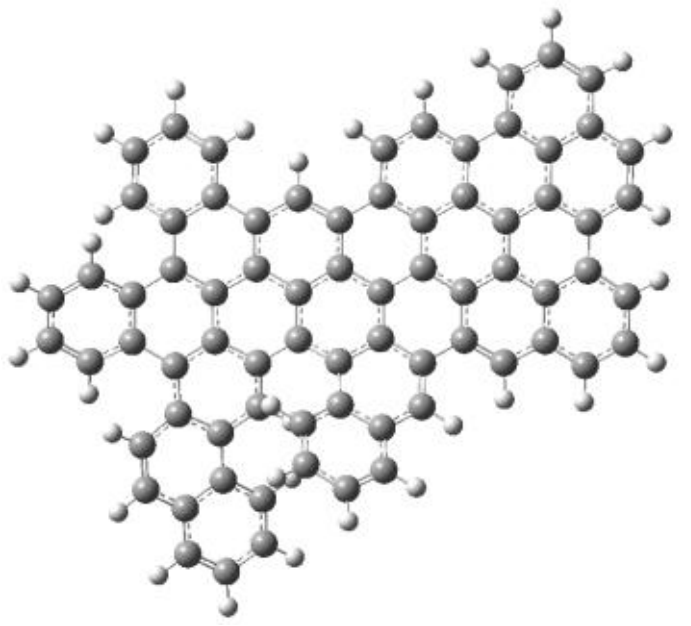

10

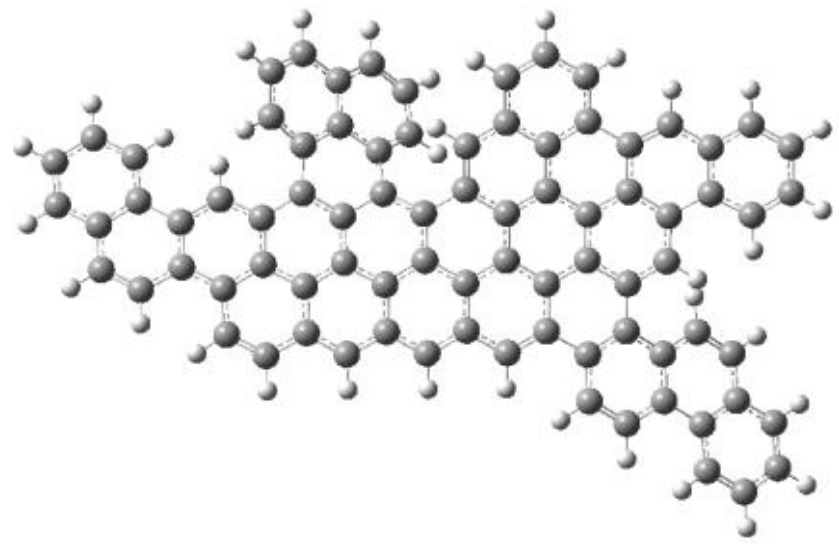

11

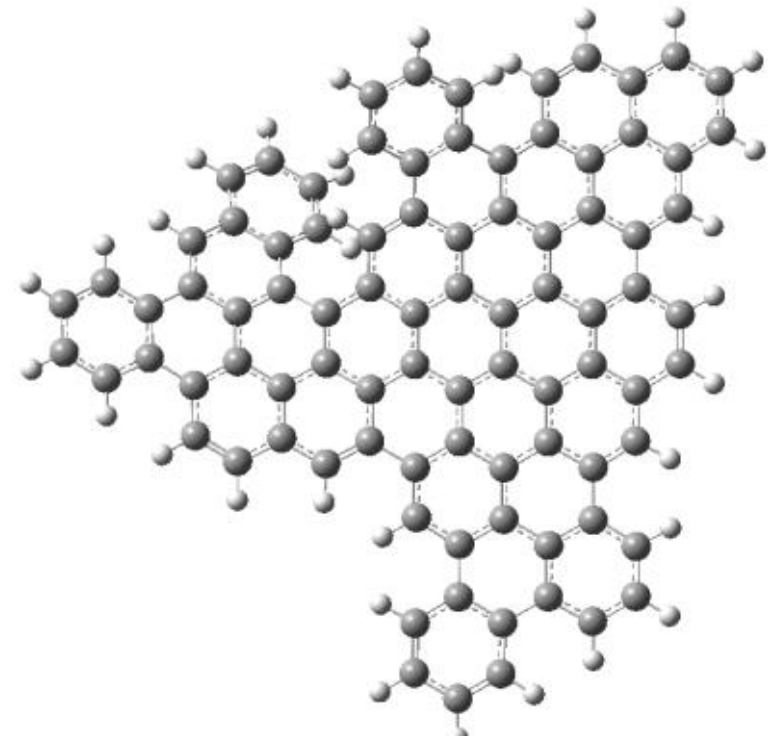

12

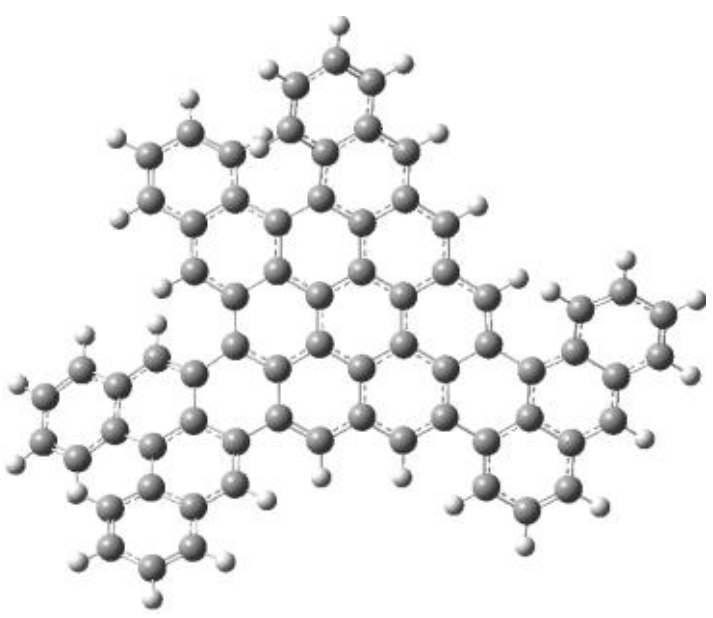

13

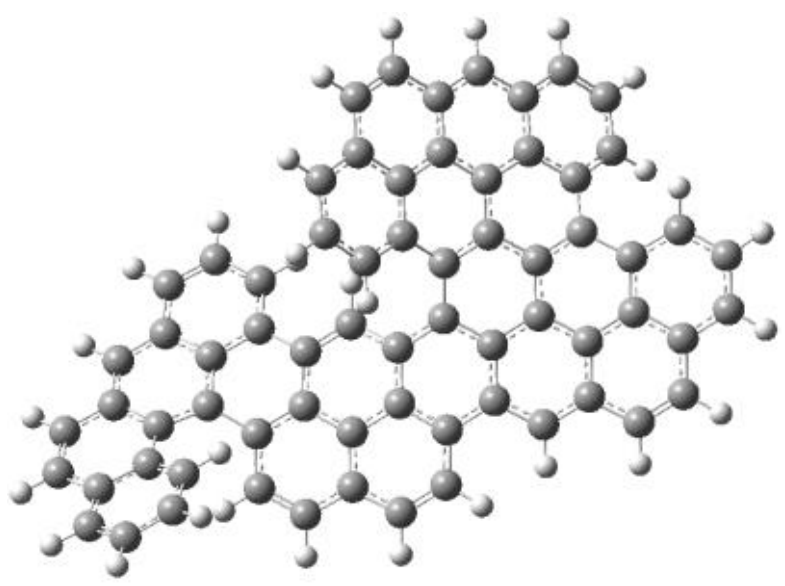

14

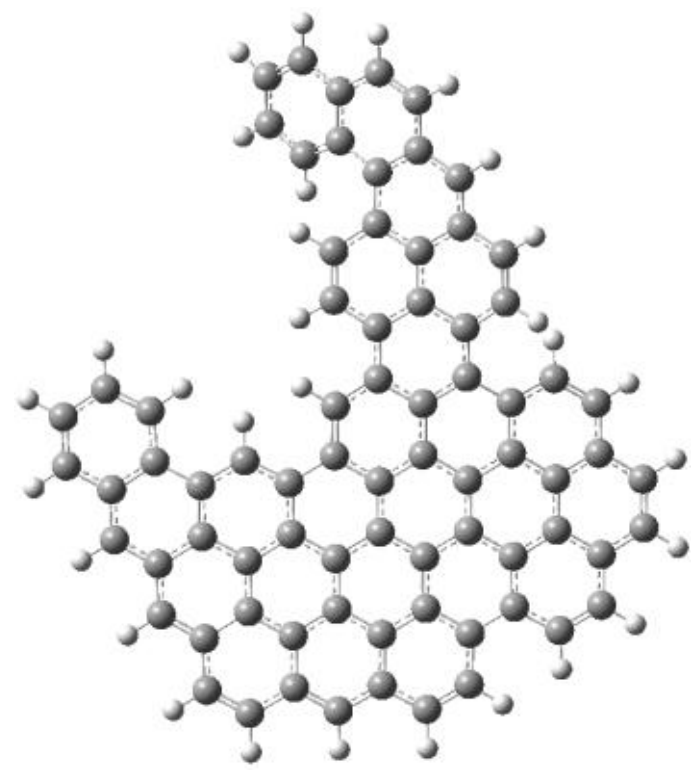

15

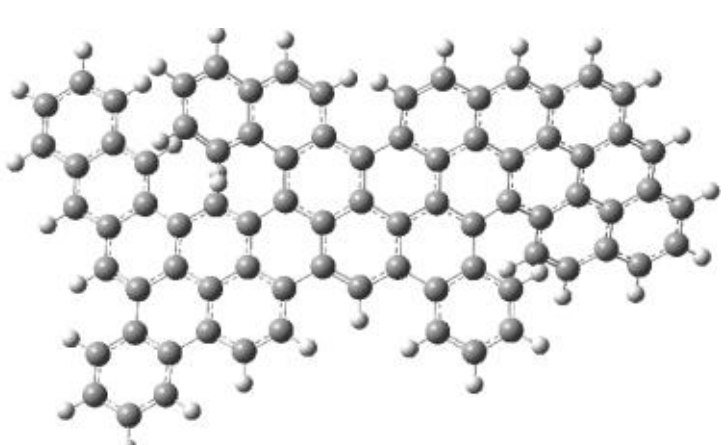

16

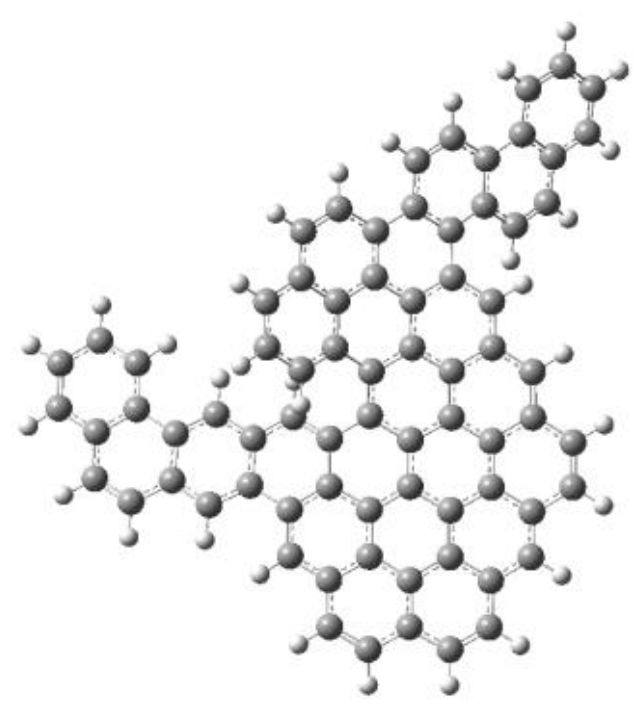

17

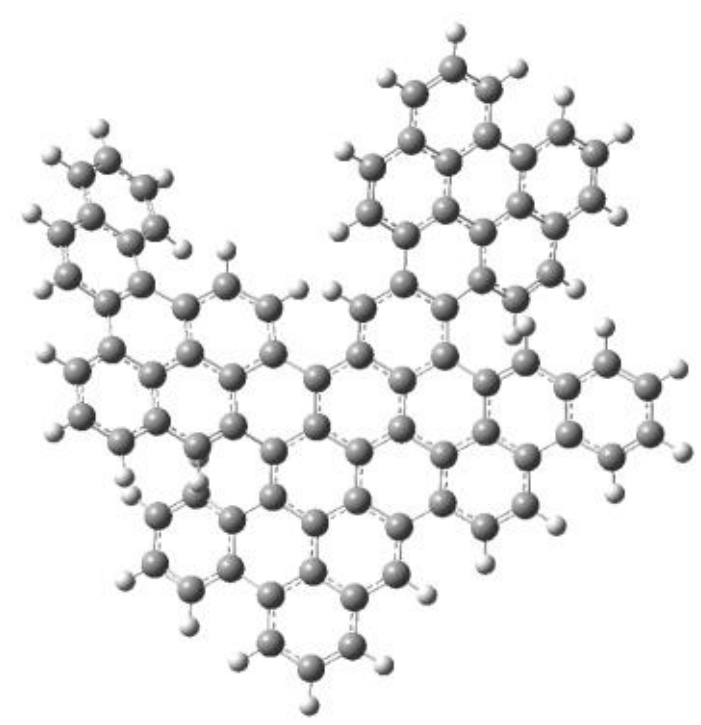

18

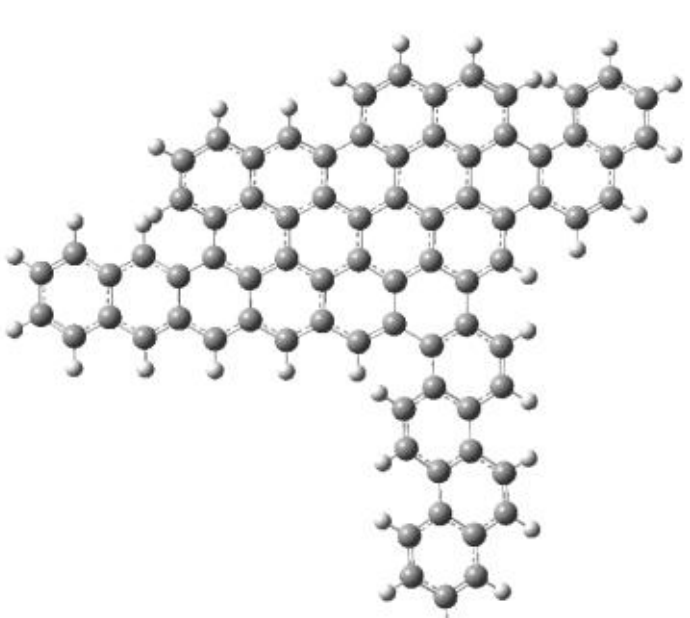

19

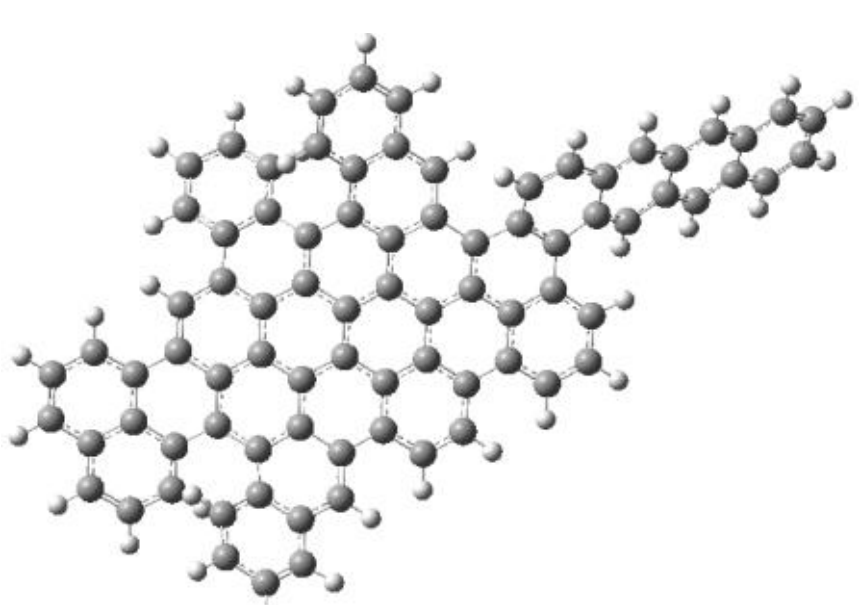

20

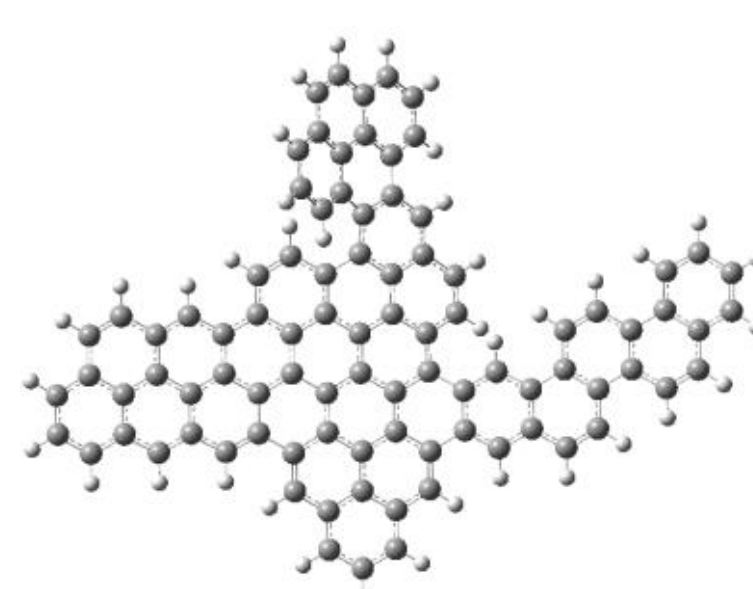

21

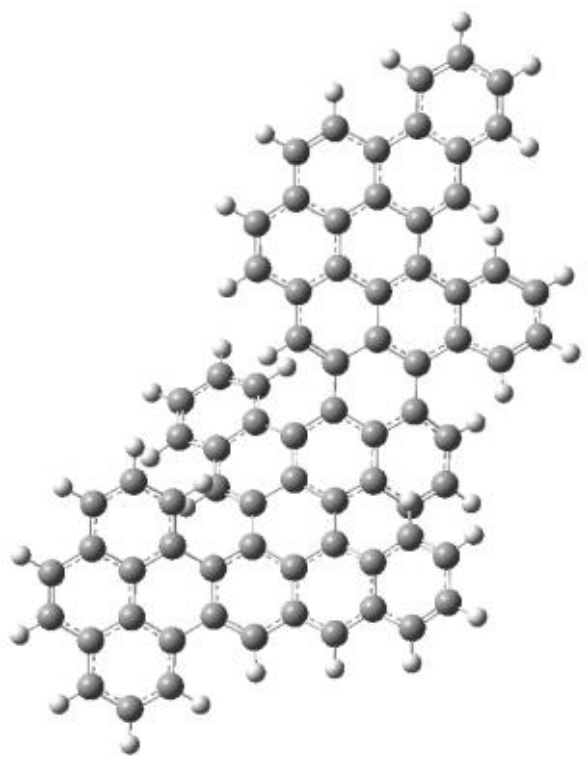

22

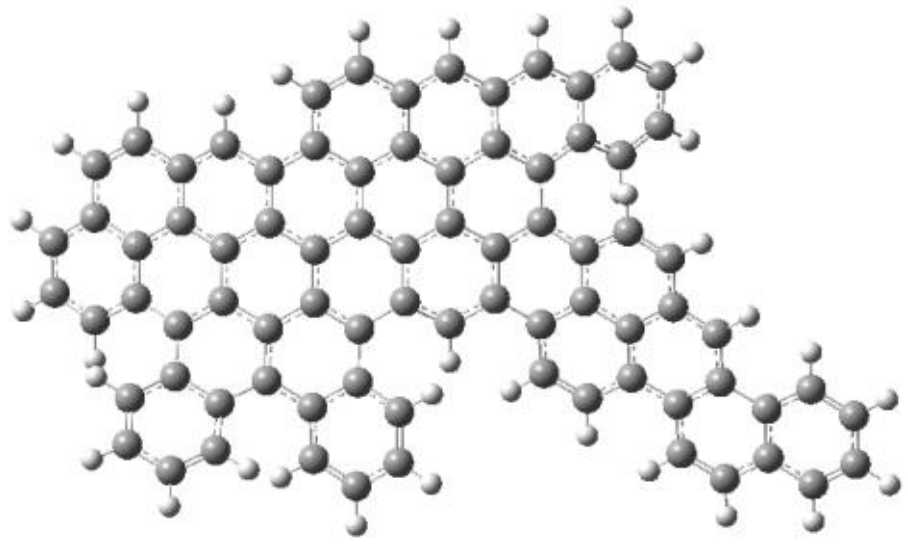

23

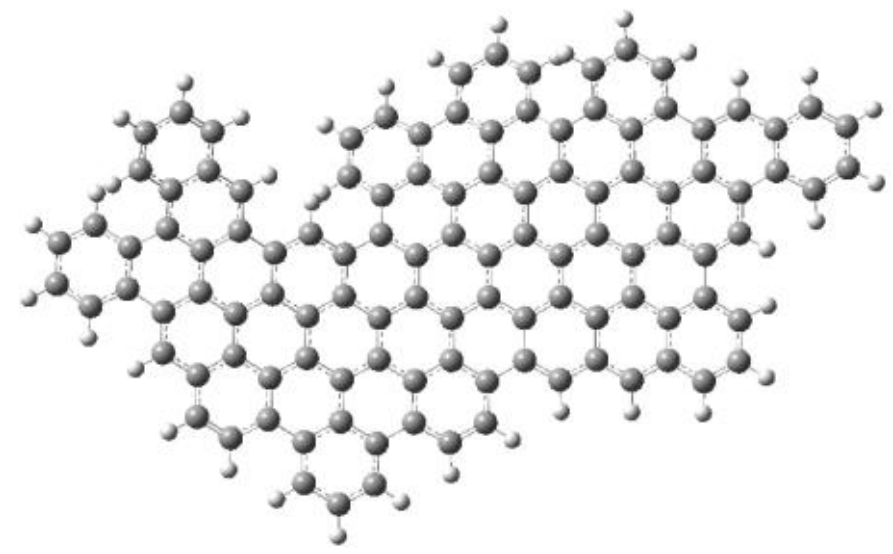

24

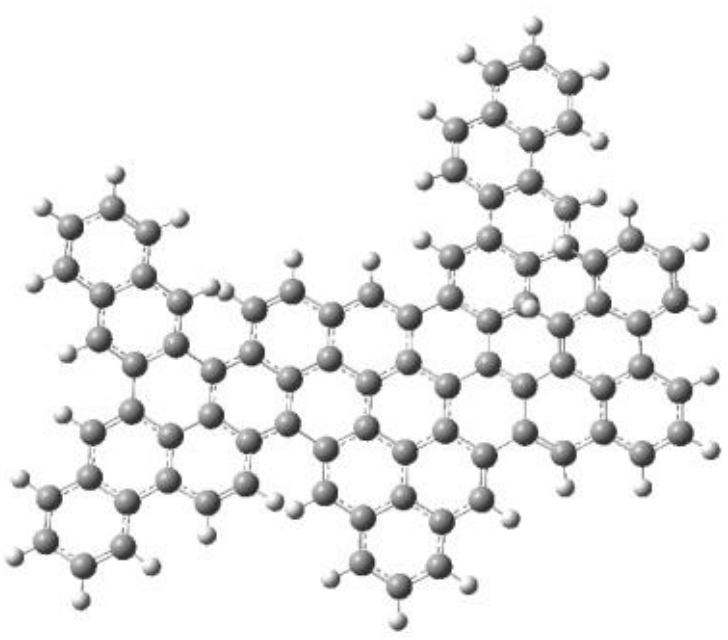

25

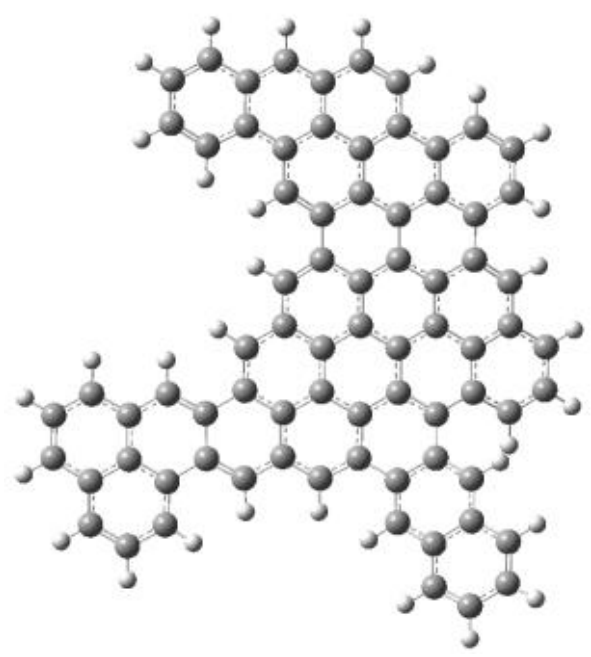

26

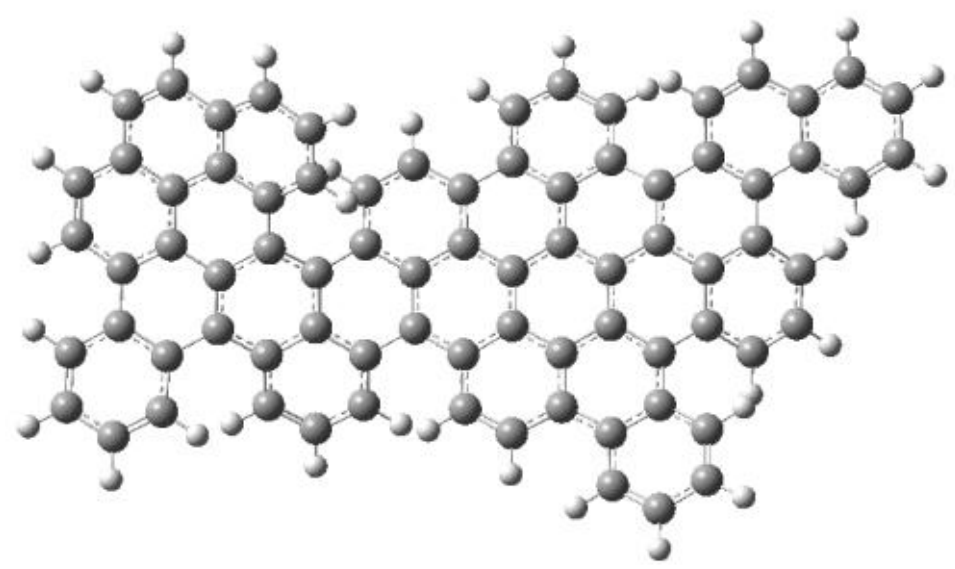

27

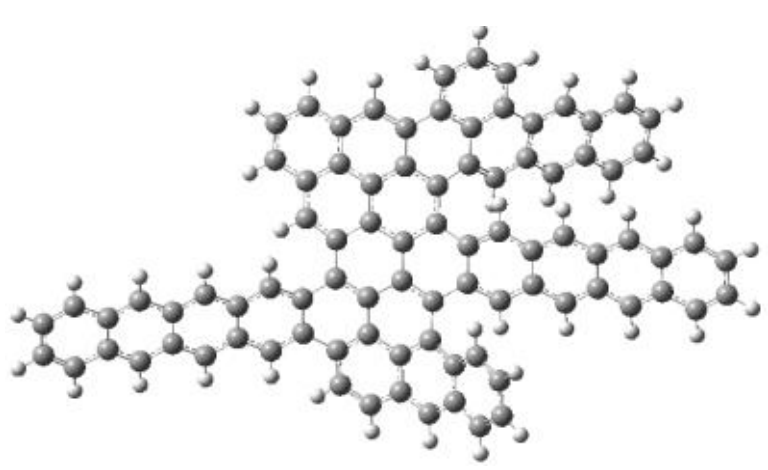

28

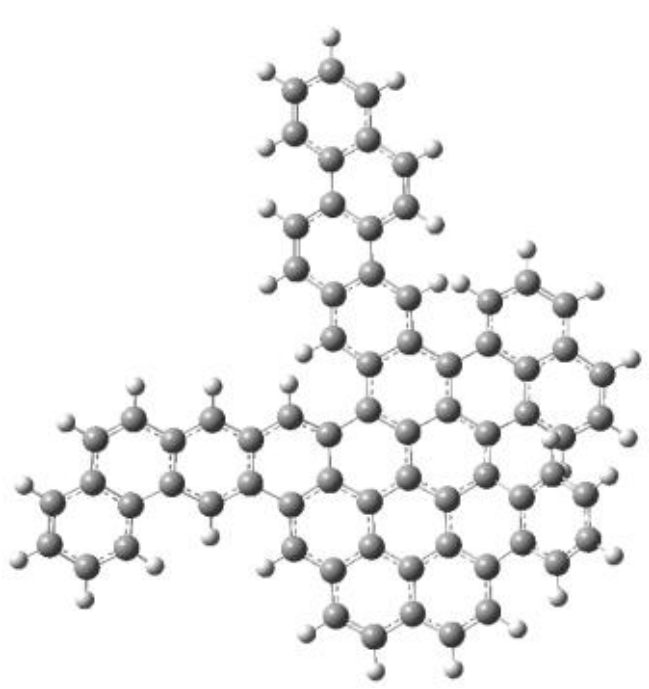

29

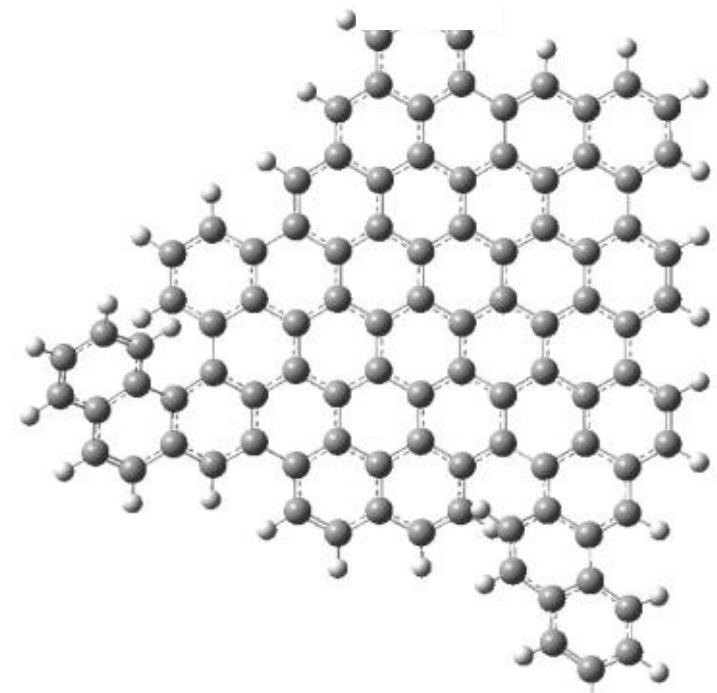

30

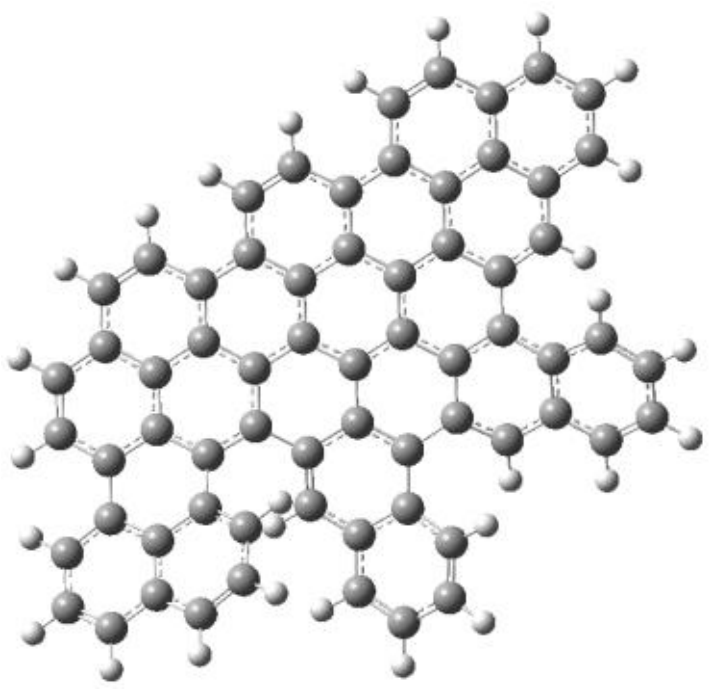

31

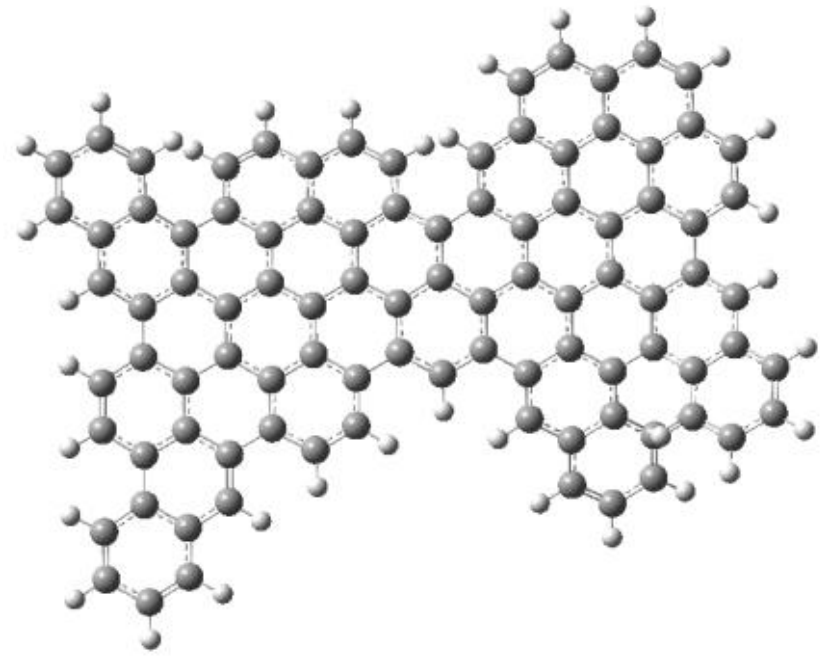

32

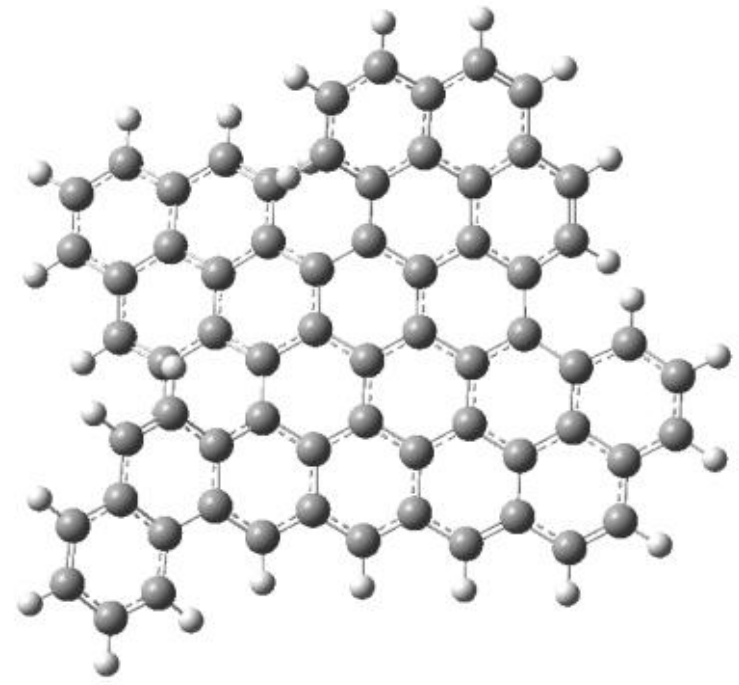

33

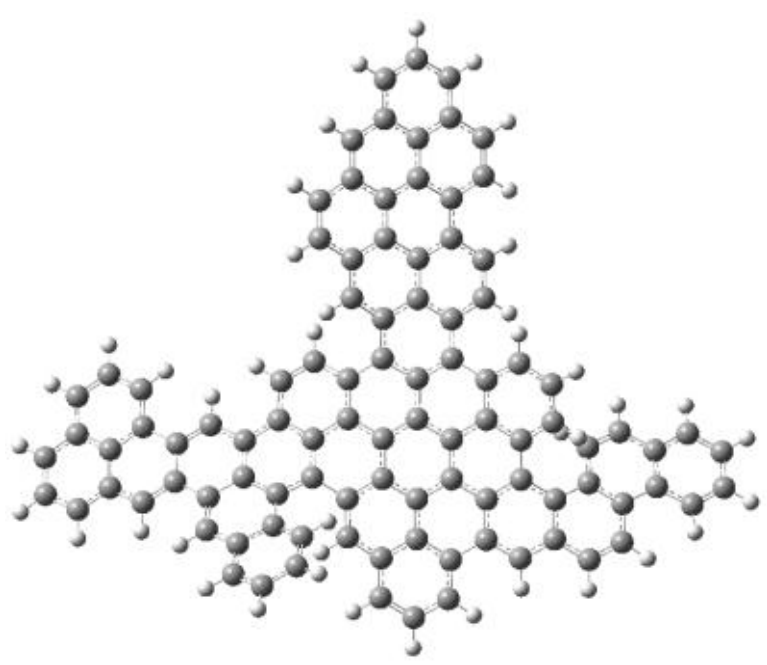

34

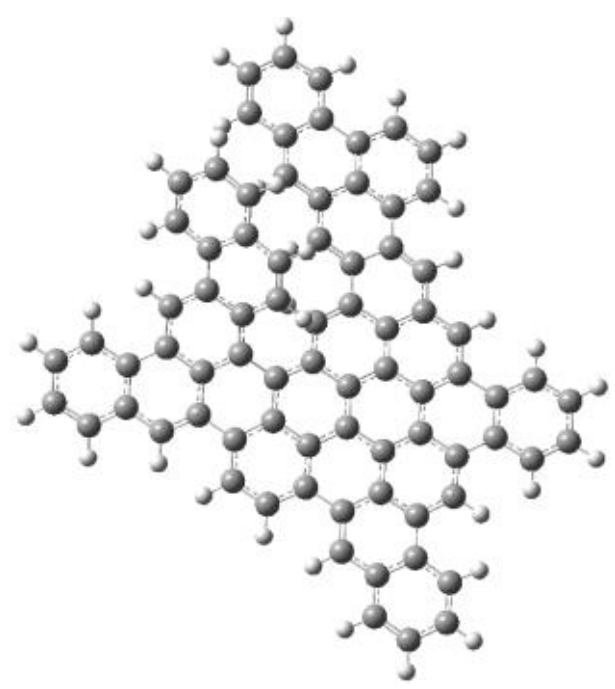

35

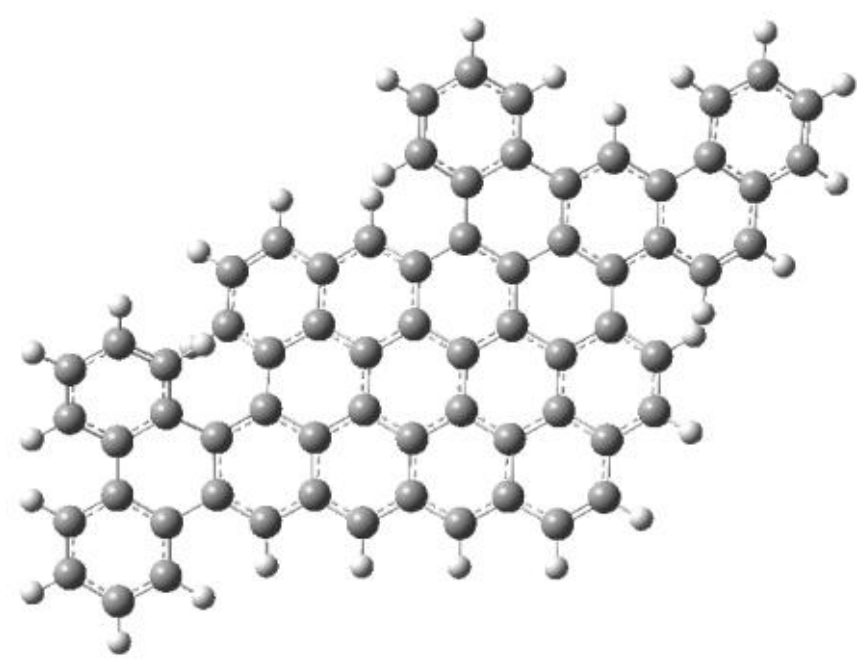

36

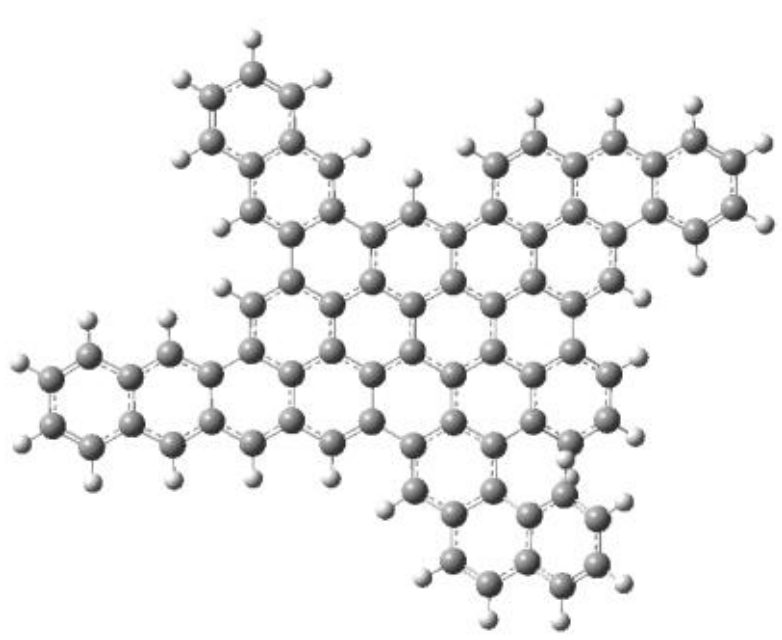

37

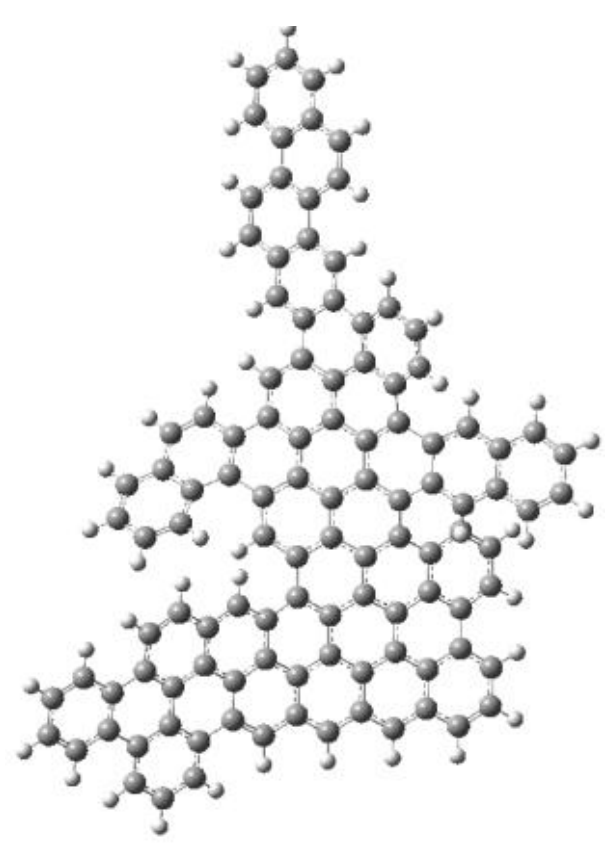

38

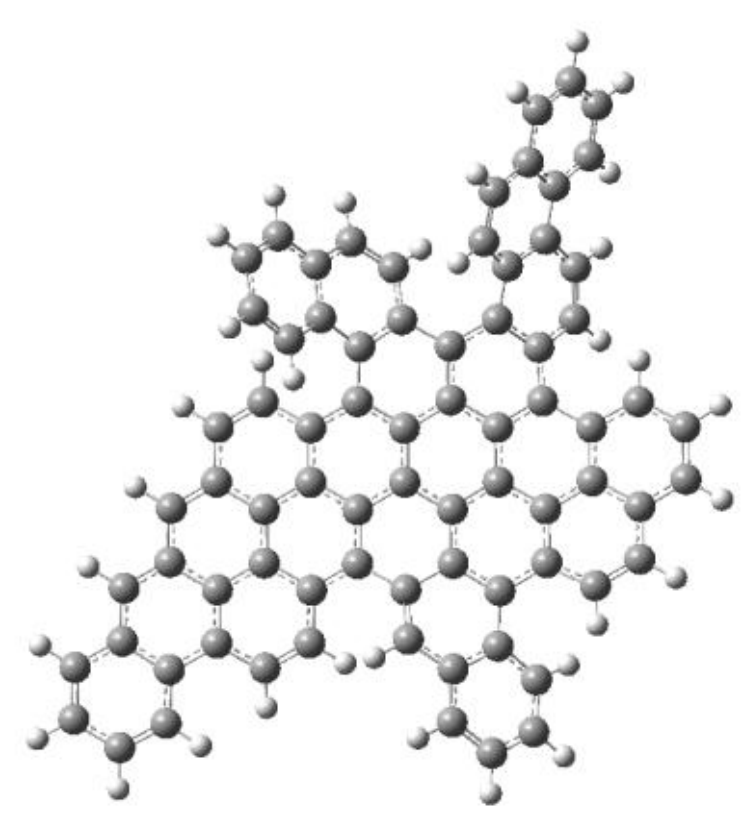

39

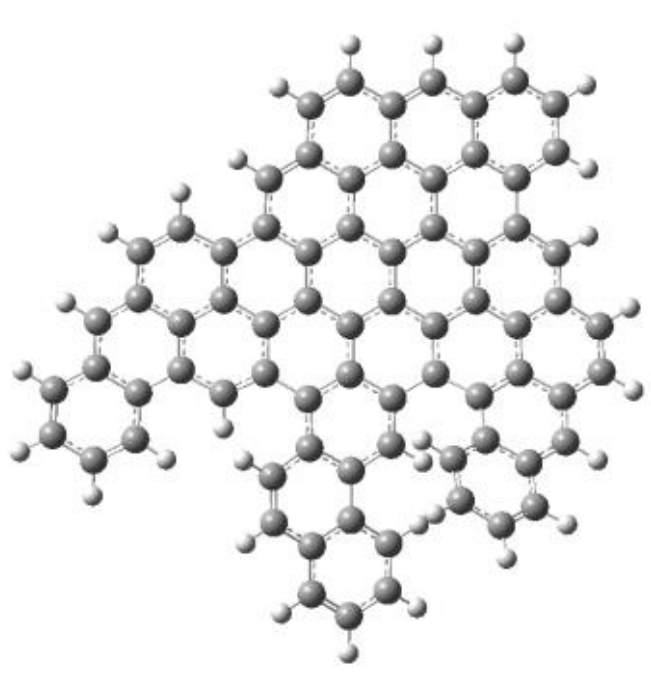

40

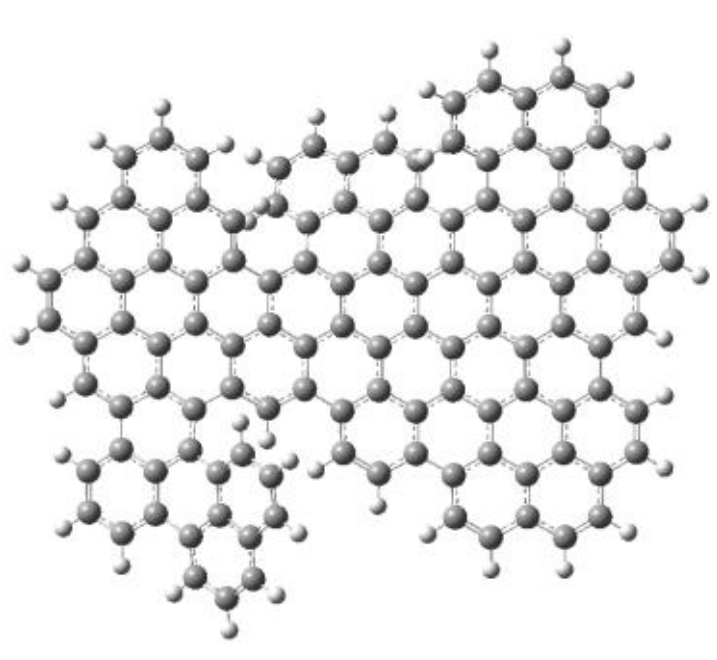

41

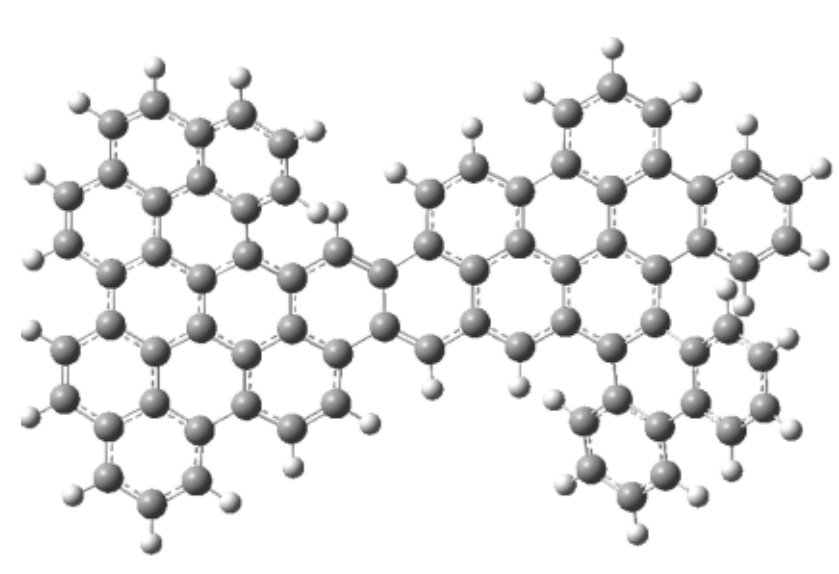

42

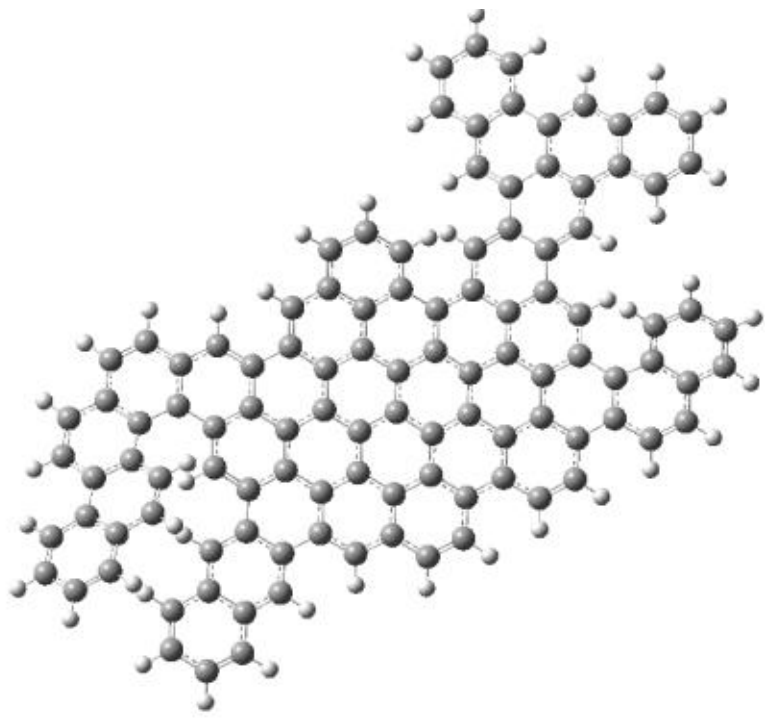

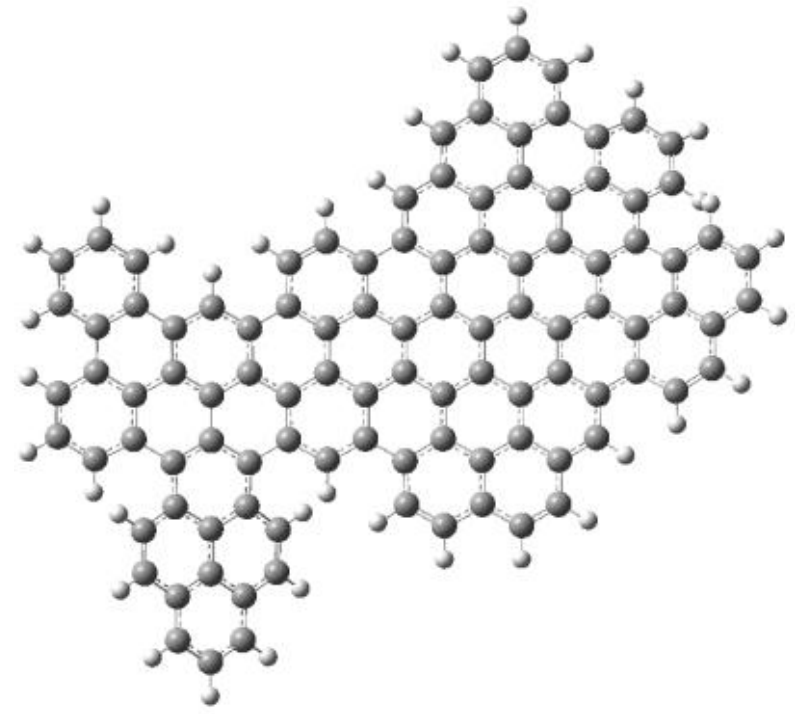

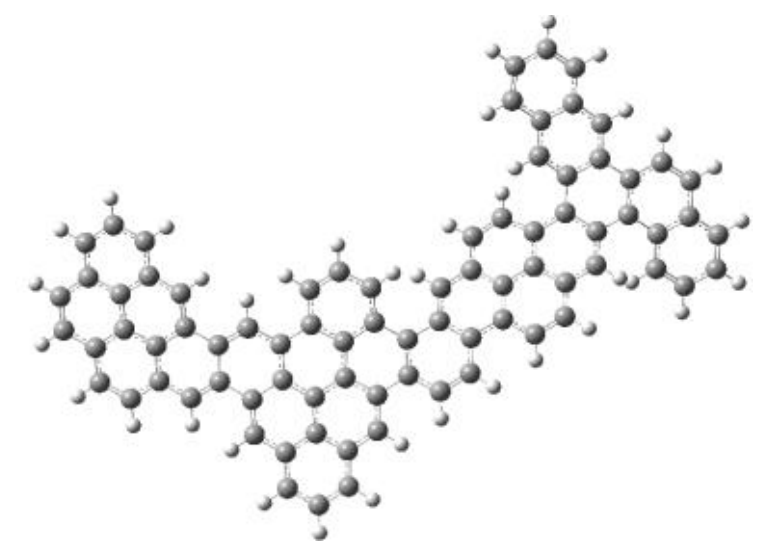

43 **44** **45**

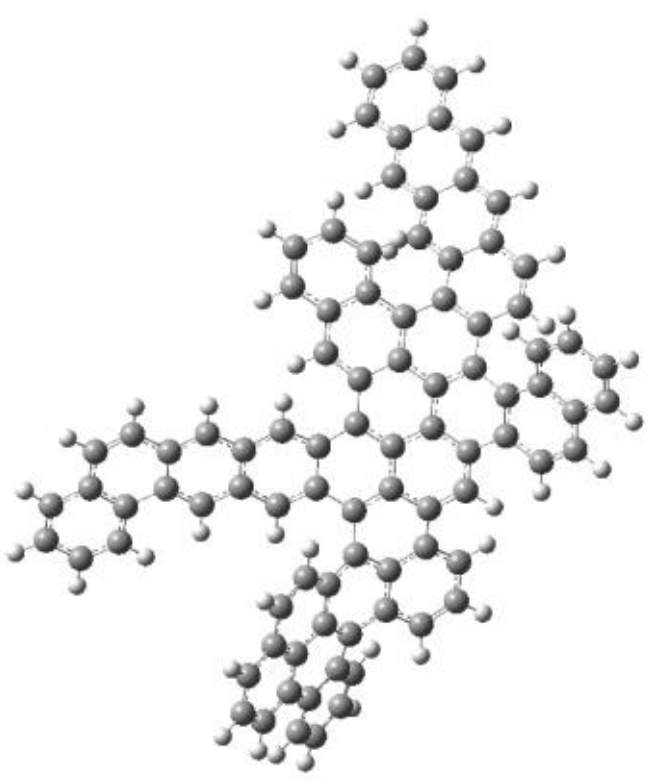

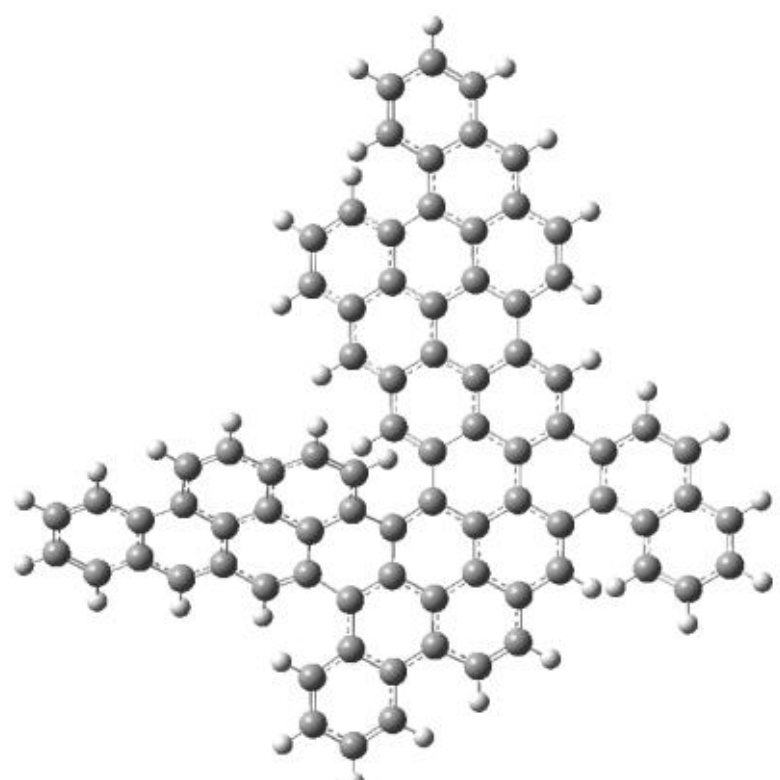

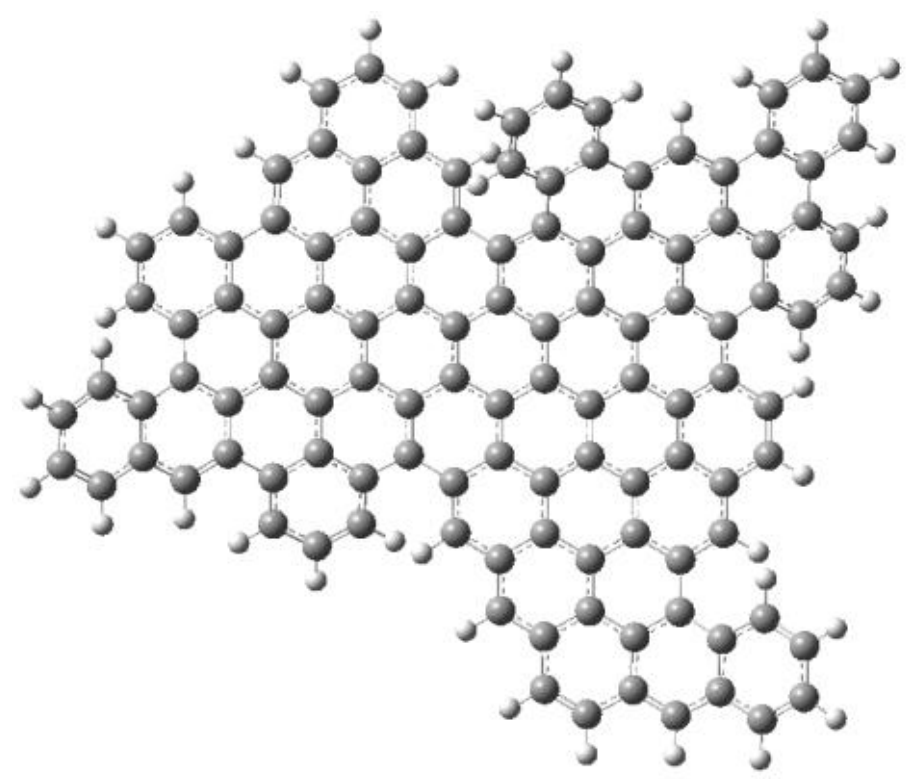

46 **47** **48**

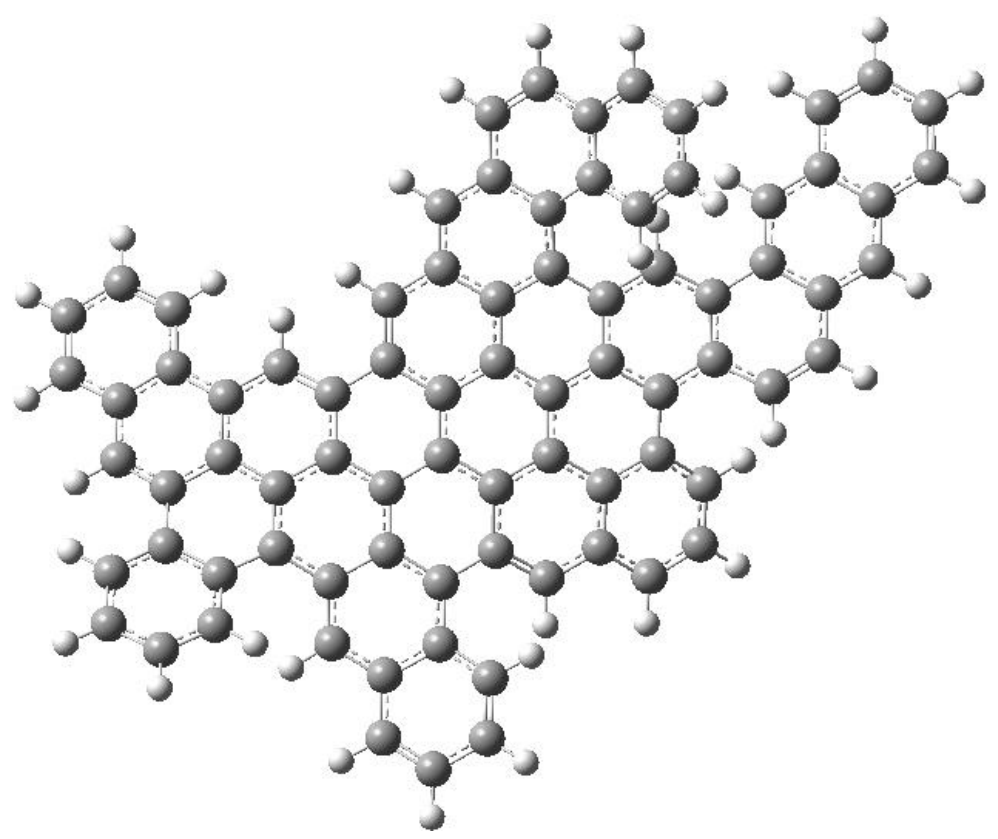

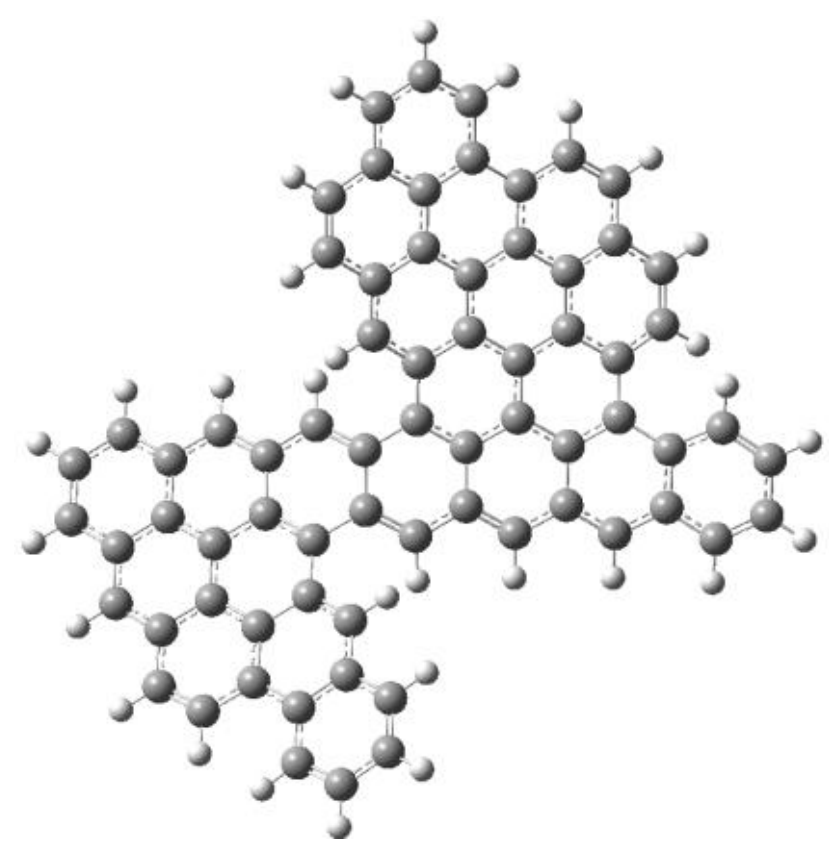

49 **50**

Figure S2: Ground-state optimized geometries of the top 50 NIR-emissive graphitic structures calculated at the B3LYP/def2-SVP level of theory.
